# Metabolic signature of COVID-19 progression: potential prognostic markers for severity and outcome

**DOI:** 10.1007/s11306-025-02264-w

**Published:** 2025-05-21

**Authors:** Hien Thi Thu Nguyen, Malene Pontoppidan Stoico, Vang Quy Le, Jakob Holm Dalsgaard Thomsen, Kasper Bygum Krarup, Karoline Assifuah Kristjansen, Inge Søkilde Pedersen, Henrik Bygum Krarup

**Affiliations:** 1 Department of Molecular Diagnostics, Aalborg University Hospital, Reberbansgade 15, 9000 Aalborg, Denmark; 2 Department of Clinical Medicine, Aalborg University, Aalborg, Denmark; 3 NOVODAN ApS, Aalborg, Denmark; 4 Department of Geriatrics, Aalborg University Hospital, Aalborg, Denmark; 5 Department of Clinical Medicine, Aarhus University, Aarhus, Denmark

**Keywords:** COVID-19, Severity and outcome prediction models, Prognostic markers, Metabolomics, NMR

## Abstract

**Introduction:**

There are significant challenges remain in accurately categorizing the risk of severe acute respiratory syndrome coronavirus 2 (SARS-CoV-2) patients.

**Objectives:**

We used an untargeted 1H NMR-based metabolomics to assess the metabolomic changes in serum samples from a Danish cohort of 106 COVID-19-infected patients with mild to fatal disease courses and from patients with fatal outcomes from other diseases.

**Methods:**

In total, 240 serum samples were used for this study. We used the data for multiple analyses (1) to construct a predictive model for disease severity and outcome, (2) to identify prognostic markers for subsequent disease severity and outcome, and (3) to understand the disease consequences in the metabolome and how recovery or death is reflected in the altered biological pathways.

**Results:**

Our results revealed distinct alterations in the serum metabolome that could differentiate patients with COVID-19 by severity (mild or severe) or outcome (death or survival). Using receiver operating characteristic (ROC) curve analysis and four machine learning algorithms (random forest, linear support vector machine, PLS-DA, and logistic regression), we identified two biomarker sets with relevant biological functions that predict subsequent disease severity and patient outcome. The range of these severity-associated biomarkers was equally broad and included inflammatory markers, amino acids, fluid balance, ketone bodies, glycolysis-related metabolites, lipoprotein particles, and fatty acid levels.

**Conclusions:**

Our data suggest the potential benefits of broader testing of these metabolites from newly diagnosed patients to predict which COVID-19 patients will progress to severe disease and which patients will manifest severe symptoms to minimize mortality.

**Supplementary Information:**

The online version contains supplementary material available at 10.1007/s11306-025-02264-w.

## Introduction

Severe acute respiratory syndrome coronavirus 2 (SARS-CoV-2) triggered a global pandemic and has emerged as a critical challenge to health care systems because of the need to provide intensive care to a previously inconceivable number of patients (Bennet et al., 2020). Although most patients experience very mild-to-moderate symptoms, approximately 14% of patients may develop severe symptoms, and up to 5% of patients become critically ill, with an overall fatality rate of 2.3% (Hasan et al., 2021).

Early recognition of a severe form as well as understanding changes in the biochemistry of individuals likely to survive versus individuals likely to die from COVID-19 are essential for timely triaging of patients. Currently, in clinical practice, those patients who are classified as having clinically severe or critical life-threatening infections are mainly diagnosed empirically on the basis of a set of clinical characteristics, such as respiratory rate (> 30 breaths·min^−1^), mean oxygen saturation (< 94% in the resting state), or arterial blood oxygen partial pressure/oxygen concentration (< 300 mmHg) (Word Health Organization, 2023). However, patients exhibiting these clinical manifestations have already progressed to a clinically severe phase and require immediate access to specialized intensive care; otherwise, they may die rapidly (Shen et al., 2020).

Additionally, effective therapies for severe cases of infection remain speculative, primarily due to poor understanding of the variability in individual responses to COVID-19 and the limited knowledge of the biological mechanisms involved in SARS-CoV-2 infection. Many studies have shown that severe complications in patients with COVID-19 arise through vasculopathy and coagulopathy elicited by infection rather than via the typical inflammatory responses normally observed in acute respiratory distress syndrome or cytokine release storms (Fox et al., 2020; Grobler et al., 2020; Leisman et al., 2020; Xiao et al., 2021; Zheng et al., 2020). Other mechanisms appear to be related to interferon responses (Arunachalam et al., 2020; Hadjadj et al., 2020). However, many of these studies have been published without clear details (Knight et al., 2020), and significant challenges remain in accurately categorizing the risk of SARS-CoV-2 patients.

Therefore, it is critical to develop new approaches to assess which cases will likely become clinically severe and which cases will likely survive or die from COVID-19. Metabolic alterations that are intensified with disease severity might help in the early recognition of severe forms. Recently, an increasing number of studies have applied metabolomic strategies to investigate COVID-19. Most studies have shown that disruption of lipid metabolism (López-Hernández et al., 2021; Thomas et al., 2020), tryptophan metabolism in relation to inflammation (Ansone et al., 2021; Bi et al., 2025; Blasco et al., 2020; López-Hernández et al., 2021) and changes in pyrimidine metabolism (Blasco et al., 2020) are metabolic features of COVID-19 patients compared with controls. However, many of these studies were performed using small cohorts, and most of them compared patients with healthy controls (Barberis et al., 2020; Blasco et al., 2020; Bruzzone et al., 2020; Shen et al., 2020; Song et al., 2020; Thomas et al., 2020); only a few reports have evaluated the metabolic differences between mild and severe cases (Shen et al., 2020; Song et al., 2020; Su et al., 2020). A recent publication identified metabolomic differences across four different clinical stages (Valdés et al., 2022) and considered aspects such as severity. There is a paucity of data regarding metabolic alterations among COVID-19 patients with very severe disease, and the biological mechanisms involved are only partially understood.

In this study, our work focused on the following question: Can the metabolome be used to differentiate patients with COVID-19 in terms of either severity (mild or severe) or outcome (death or survival) and among patients with fatal outcomes (COVID-19 or other diseases)? Thus, we explored the serum metabolome of non-COVID-19 controls, COVID-19 patients at three different stages, and patients who died from other diseases using NMR-based metabolomics. COVID-19 patients were stratified on the basis of their clinical evolution into mild or moderate disease (not requiring hospitalization), severe disease (requiring hospitalization), and critical disease (requiring hospitalization) with fatal outcomes. In addition, follow-up samples were obtained from hospitalized patients up to 7 months after hospital admission to investigate the disease effects on the metabolome and how recovery or death is reflected in the biological pathways. The final goal of the current work is to identify biomarkers that will increase our understanding of how COVID-19 infection evolves and improve our ability to predict how a patient will progress on the basis of the metabolite profile of serum samples obtained at an early stage of infection.

## Materials and methods

### Ethical approval

The study was approved by The North Denmark Region Committee on Health Research Ethics (Protocol N-20200031). The investigation did not interfere with patient treatment. The committee provided exemption for written informed consent for the patients, and all healthy controls provided written informed consent.

### Patients and controls

Serum samples were collected from adult patients admitted to Aalborg University Hospital, Aalborg, Denmark, who were diagnosed with SARS-CoV-2 infection during the first and second waves of the pandemic from March 2020 to October 2020. These samples were stored in the Bio- and Genome Bank Denmark [Danish Covid-19 Biobank (D19B)].

COVID-19 diagnosis was based on SARS-CoV-2 detection via real-time reverse transcription polymerase chain reaction of a nasopharyngeal swab or bronchoalveolar lavage. The classification of disease severity was based on the World Health Organization ‘COVID-19 Clinical Management: Living Guidance’ (Jan 25, 2021).

A total of 37 COVID-19-negative (HEA) patients (people with COVID-like symptoms but test negative by PCR), 106 COVID-19 patients and 17 patients with fatal outcomes from other diseases (Other-D) were included. Among the 106 COVID-19 patients, 32 had mild or moderate disease and did not require hospital admission (COV-M); 38 had severe disease, requiring hospitalization, and with recovery outcome (COV-L); and 36 had severe disease, requiring hospitalization, and with fatal outcome (COV-D).

**Fig. 1:**
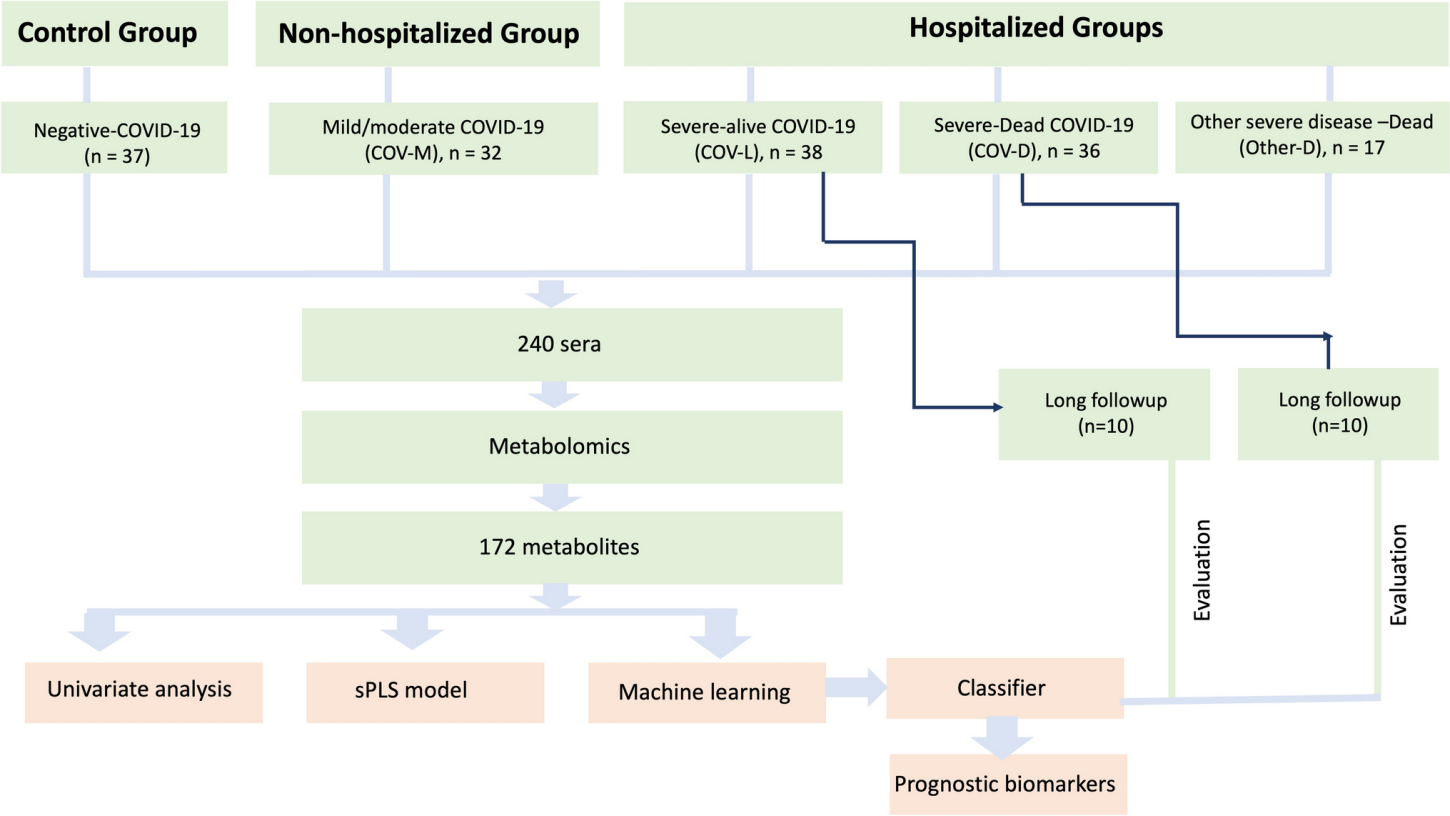
Flow chart of the study

Among 74 hospitalized patients, 20 patients with at least 2 samples each obtained at different time points were processed with the aim of evaluating metabolic biomarkers of illness recovery or death. In total, 240 serum samples from COVID-19 patients, patients with other fatal diseases, and healthy controls were used for this study (Fig. 1).

### NMR-based metabolomics

Around 9 ml of peripheral venous blood was collected from patients and controls. The blood was left to clot at room temperature for 1 h, then centrifuged at 2000×*g* for 10 min at 25 °C. The serum was separated and stored at − 80 °C.

The lipid and metabolic measures were quantified by high‐throughput NMR metabolomics service (Nightingale Health Ltd., Helsinki, Finland). The NMR based measurements were conducted from 350 μl of stored serum samples using a 600 MHz Bruker AVANCE III HD NMR spectrometer (Bruker BioSpin, Switzerland) with automated sample changer and cryoprobe. This NMR platform enables the simultaneous quantification of routine lipids, lipoprotein subclass profiling with lipid concentrations across 14 subclasses, fatty acid composition, and a range of low-molecular-weight metabolites, such as amino acids, ketone bodies, and gluconeogenesis-related metabolites, all measured in molar concentration units. Biomarkers were independently quantified for each serum sample, without reference to data from other samples within the same well plate or cohort. The average success rate for metabolite quantification was 99%. Previous review has detailed the technological aspects and epidemiological applications of the Nightingale NMR platform (Würtz et al., 2017). The process underlying this NMR metabolomics technology has received regulatory approval (CE).

Complete results for all the 172 measures quantified by the Nightingale NMR platform are reported in Table S0.

### Statistical analysis

The statistical analysis was performed according to two different experimental designs.

In the first experimental design, samples collected from patients at hospital admission were analyzed. Principal component analysis (PCA) was used to obtain a wider view of the metabolomics changes during the course of disease in COVID-19 patients as well as the metabolic variance between the non-COVID-19 control group (HEA) and patient groups. Heatmap representations were obtained, and all groups were compared via nonparametric ANOVA (Fisher’s LDL test).

In the second experimental design, the random forest algorithm was used to develop classification models and predict patient outcomes. First, the recursive feature elimination (RFE) method was applied to recursively remove features to identify a subset of variables that contribute most to predicting the outcome. The most important feature predicted by RFE was subsequently used to classify the model and predict patient outcomes. The data were divided randomly into a training set and a test set, with 80% of the patients in the training set and 20% in the test set. The hyperparameters (i.e., max_depth, max_features and n_estimators) were optimized using a cross-validation search. The best set of parameters was selected using a scoring system that combined accuracy, F1 score and precision. The combination of hyperparameters that resulted in the highest score of the scoring criteria was selected and used to train the model. The test dataset was then used to test the prediction accuracy. The results were then summarized in a confusion matrix. The features (i.e., columns of data) that had the greatest influence on the outcome were sorted as produced by the importance list.

Two-dimensional spare partial least squares discriminant analysis (2-D sPLS-DA) score plots were used to compare serum metabolite data across and between study groups. Fivefold cross-validation was chosen to evaluate the classification performance of the sPLS-DA model. For this model, the error rate is calculated to evaluate the performance.

The second experimental design consisted of assessing the strength of the associations between individual metabolites and the stages/statuses of patients with COVID-19. A receiver operating characteristic (ROC) curve analysis was performed to generate a ROC curve that was subsequently used to determine the area under the curve (AUC) and the 95% confidence interval. The ROC curve summarizes the sensitivity and specificity of a single feature to classify data accurately, which can then be used to compare the overall accuracy of different biomarkers. Four machine learning algorithms (random forest, linear support vector machine, PLS-DA, and logistic regression) were then applied to develop prediction models for the progression of COVID-19 on the basis of the identified metabolomic biomarkers. Finally, a permutation test was used to indicate whether the specific classification model was superior to random classifiers.

Furthermore, the analysis and comparison of serum samples from patients belonging to the hospitalized groups at two different time points (at hospital admission and at the end of treatment) were performed with the aim of evaluating metabolic biomarkers of illness recovery or death.

All univariate and multivariate statistical analyses, biomarker design and validation were performed using the MetaboAnalystR 4.0 R package (https://github.com/biocyberman/MetaboAnalystR/tree/dev) and R package.

## Results

### Patient baseline

A total of 160 participants were involved in this research study, 37 of whom were COVID-19 negative (HEA) (median age: 56.6 years [IQR, 15.3 years]), 106 were COVID-19 patients (median age: 66.1 years [IQR, 18.9 years]), and 17 were patients with fatal outcomes from other diseases (other D) (median age: 84 years [IQR, 8.7 years]). Among the total number of COVID-19 patients (106 patients), 32 had mild or moderate disease and did not require hospital admission (COV-M) (median age: 46.9 years [IQR, 12.5 years]); 38 had severe disease, requiring hospitalization but with recovery (COV-L) (median age: 67.8 years [IQR, 17.2 years]); and 36 had severe disease, requiring hospitalization and resulting in a fatal outcome (COV-D) (median age: 85.4 years [IQR, 8.4 years]). There was a greater proportion of males in the hospitalized groups, especially in the COV-D group (78%), than in the nonhospitalized groups (HEA: 46% and COV-M: 31%). The median age was also notably greater in the hospitalized and deceased groups (COV-D: 85.4 ± 8.4; and Other-D: 84 ± 8.7 years) than in the other groups (56.6, 46.9 and 67.8 years for HEA, COV-M and COV-L, respectively). The mean number of days admitted to the hospital after disease onset was greater in the COV-L group (24.3 ± 43.4 days) than in the COV-D group (13 ± 13.3 days) (Supplementary Table S1).

3.2 Models to distinguish COVID-19-negative controls from COVID-19-positive patients at different stages of the disease, as well as from patients who died due to other, non-COVID-19-related illnesses.

**Fig. 2:**
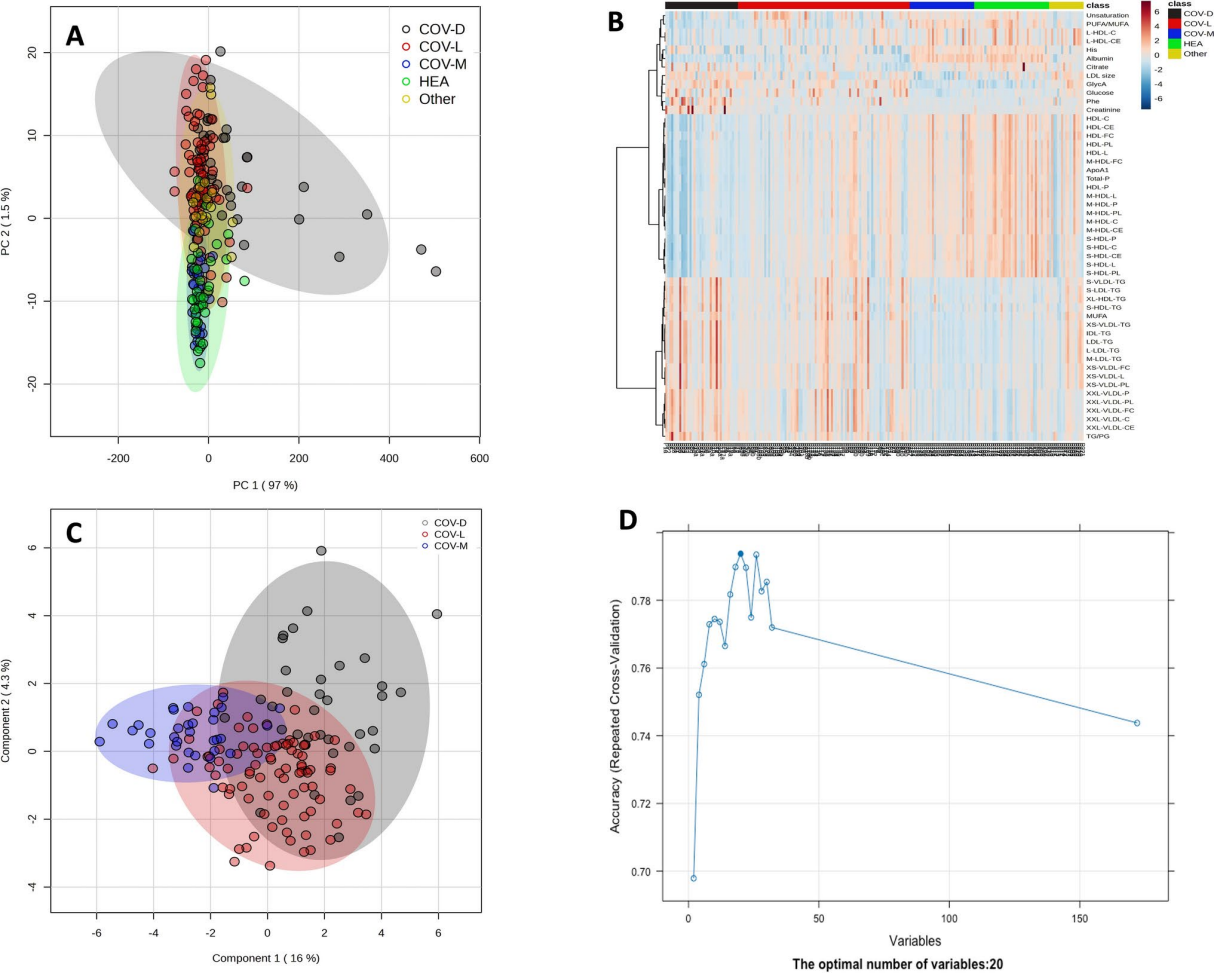
Models to distinguish COVID-19-negative controls from COVID-19-positive patients at different stages of the disease, as well as from patients who died due to other, non-COVID-19-related illnesses. **A** PCA showing the discrimination between the non-COVID-19 control group (HEA) (green), the COVID-19 patient groups at different stages, patients with mild/or moderate disease who did not require hospital admission (COV-M) (blue), patients with severe disease who required hospitalization and recovered (COV-L) (red), patients with severe disease who required hospitalization and died (COV-D) (black), and patients who died due to other, non-COVID-19-related illnesses (Other) (Orange). **B** Heatmap of 50 selected metabolites with a false discovery rate (FDR) < 0.05. **C** sPLS score plot of COV-M (blue) vs. COV-L (red) vs. COV-D (black) patients. **D** Recursive feature elimination (RFE) analysis showing the specific number of variables that contribute most to predicting the outcome of COVID-19 patients

To better understand the metabolomic changes throughout the progression of COVID-19—and to explore the metabolic differences among the non-COVID-19 control group, patients with fatal outcomes from other diseases, and various COVID-19 patient subgroups—a PCA model was developed. PCA analysis indicated that patients with mild or moderate COVID-19, who did not require hospitalization, clustered closer to the non-COVID-19 control group. In contrast, COVID-19 patients with severe disease who were hospitalized but survived showed greater similarity to non-COVID-19 patients who were hospitalized and died (Fig. 2A, B). Notably, COVID-19 patients with severe disease who were hospitalized and subsequently died exhibited distinct metabolomic profiles compared to all other groups. The ANOVA with Fisher’s LDL test revealed 118 metabolites that exhibited significantly altered abundance (Supplementary Table S2). The heatmap representation of these altered metabolites showed similar results as those obtained using PCA. Patients with mild or moderate COVID-19 showed greater similarity to the non-COVID-19 control group, while those with severe disease who survived were more closely aligned with the group of deceased patients with other illnesses. In other words, hospitalized patients exhibited distinct metabolomic profiles compared to nonhospitalized individuals (Fig. 2B). Moreover, COVID-19 patients with severe disease who were hospitalized and later died exhibited unique metabolomic signatures that set them apart from all other groups.

In the next step, the RFE method was applied to identify the subset of variables that contributed the most to predicting the outcome of COVID-19 patients. RFE analysis revealed that the optimal number of variables for classification of the three stages of COVID-19 was 20 (Fig. 2D).

**Fig. 3:**
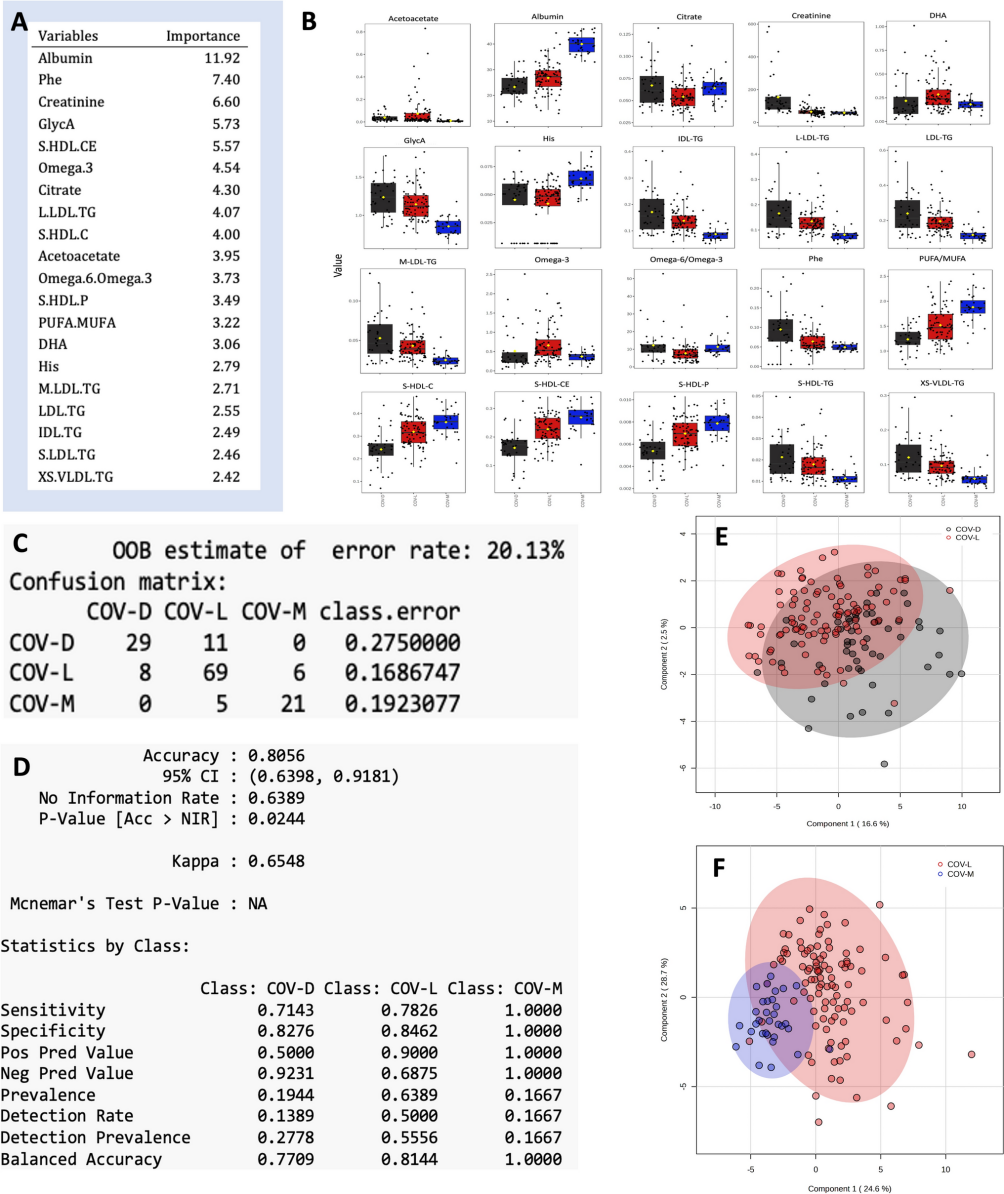
Variable importance for different stages of COVID-19 and the RF model for classification and validation for different stages of COVID-19. **A** The top twenty metabolites responsible for differentiating COVID-19 severity according to random forest (RF) analysis. **B** Biochemical shifts in these twenty metabolites associated with the severity of COVID-19. Prediction model metrics using 20 factors in both classification (**C**) and validation (**D**) for different stages of COVID-19. **E** sPLS score plot of COV-L (red) vs. COV-D (black). **F** sPLS score plot of COV-L (red) vs. COV-M (blue)

On the basis of the random forest analysis, the top twenty metabolites responsible for the differentiation of COVID-19 severity were albumin, phenylalanine, creatinine, glycoprotein acetyls (GlyA), cholesteryl esters in small HDL (S-HDL-CE), omega-3, citrate, triglycerides in large LDL (L-LDL-TG), cholesterol in small HDL (S-HDL-C), acetoacetate, the ratio of omega-6 fatty acids to omega-3 fatty acids (omega-6/omega-3), the concentration of small HDL particles (S-HDL-P), the ratio of polyunsaturated fatty acids to monounsaturated fatty acids (PUFAs/MUFAs), docosahexaenoic acid (DHA), histidine (His), triglycerides in medium LDL (M-LDL-TG), triglycerides in LDL (LDL-TG), triglycerides in IDL (IDL-TG), triglycerides in small LDL (S-LDL-TG) and triglycerides in very small VLDL (XS-VLDL-TG) (Fig. 3A). Among these 20 metabolites, all except citrate, DHA, histidine, omega-3, and the omega-6/omega-3 ratio showed biochemical shifts associated with COVID-19 severity. The levels of albumin, citrate, histidine, PUFA/MUFA, S-HDL-C, S-HDL-CE, and S-HDL-P were significantly lower in the hospitalized groups (COV-L and COV-D) than in the nonhospitalized group (COV-M) (p < 0.05 for all). In contrast, acetoacetate, creatinine, GlycA, IDL-TG, L-LDL-TG, LDL-TG, M-LDL-TG, phenylalanine, S-LDL-TG, and XS-VLDL-TG levels were increased abnormally in COV-D patients (Fig. 3B).

The twenty variables with the most significant values identified by random forest (RF) were then used by machine learning models to classify and validate different COVID-19 stages. The RF model shows differential clustering among stages of COVID-19, with 80% accuracy in both classification and validation (Fig. 3C and D). For each group in the validation analysis, the RF model could predict 77%, 81% and 100% accuracy; 71%, 78% and 100% sensitivity; and 83%, 84% and 100% specificity for the severe-dead, severe-alive, and mild/moderate groups, respectively (Fig. 3D).

Similar results were obtained for the sPLS analysis. Clear differences in the patient metabolome were noted between the COV-M group and the groups requiring hospitalization (COV-L and COV-D) as well as between the COV-L group and the COV-D group (Figs. 2C; 3E, and F). The sPLS-DA model for COV-D vs. COV-L and COV-L vs. COV-M show separation, with overall cross-validated accuracies of 79% and 90%, respectively (data not shown). sPLS-DA cannot distinguish COVID-19 patients with mild to moderate disease from non-COVID-19 controls on the basis of serum metabolites (data not shown).

### Early biomarkers for the prognosis/prediction of COVID-19 severity

A total of 172 metabolites were identified in the serum of the studied patients (Supplementary Table S0).

#### Biomarkers for the prognosis/prediction of those who will develop mild vs. severe COVID‑19 symptoms

To assess the strength of the associations between individual metabolites and the hospital status of COVID-19 patients, a classical univariate receiver operating characteristic (ROC) curve analysis was performed. Biomarker analysis using classical univariate ROC curves.

revealed that forty-nine metabolites with valid AUC values (greater than 7) were related to the hospital risk stratification of COVID-19 patients (Supplementary Table S3).

To achieve greater accuracy in distinguishing between two groups, the RFE method was applied. The RFE showed that a subset of 12 variables (biomarkers) contributed the most to predicting the hospitalization status of COVID-19 patients (data not shown).

For further exploration, the twelve metabolites with the highest AUC values were chosen to build a prediction model using four machine learning algorithms (random forests, linear support vector machine, PLS-DA and logistic regression). These metabolites include albumin, GlycA, L-LDL-TG, LDL-TG, histidine, M-LDL-TG, IDL-TG, XS-VLDL-TG, acetoacetate, glucose, S-LDL-TG and PUFA/MUFA. Among them, nine metabolites, including inflammation markers, triglycerides in small/medium/large LDL, triglycerides in IDL, ketone bodies, and glycolysis-related metabolites, demonstrated significantly higher concentrations in the hospitalized group than in the nonhospitalized group (p < 0.05), whereas the concentrations of albumin, histidine and fatty acid were significantly higher in the nonhospitalized group than in the hospitalized group (p < 0.05). The detailed information is summarized in Table 1.

**Table 1:** Biomarker set including 12 metabolites and prediction models based on these metabolite biomarkers to distinguish the hospitalized patient group (COV-L) from the nonhospitalized patient group (COV-M)

Area under the curve, p value, and log2 fold change for a set of 12 metabolite biomarkers			
Metabolites	AUC	P value	FC
Albumin	0.97	< 0.001	− 0.57566
GlycA	0.91	< 0.001	0.41865
L-LDL-TG	0.88	< 0.001	0.72487
LDL-TG	0.88	< 0.001	0.72903
His	0.88	< 0.001	− 0.61853
M-LDL-TG	0.88	< 0.001	0.77396
IDL-TG	0.86	< 0.001	0.66047
XS-VLDL-TG	0.85	< 0.001	0.70089
Acetoacetate	0.84	< 0.001	2.84
Glucose	0.84	< 0.001	0.64991
S-LDL-TG	0.83	< 0.001	0.661111
PUFA/MUFA	0.79	< 0.001	− 0.30746

Prediction models based on a set of 12 metabolites			
Algorithm	AUC	Coefficient of variation prediction	P value
PLS-DA	0.98 (0.95–1)	0.90	< 0.001
Linear support vector	0.98 (0.94–1)	0.91	< 0.001
Logistic regression	0.91 (0.81–0.98)	0.83	< 0.001
Random forest	0.98 (0.93–1)	0.92	< 0.001

*LDL* low-density lipoproteins, *IDL* intermediate-density lipoproteins, *VLDL* very-low-density lipoproteins, *L-LDL-TG* triglycerides in large LDL, *LDL-TG* triglycerides in LDL, *M-LDL-TG* triglycerides in medium LDL, *IDL-TG* triglycerides in IDL, *XS-VLDL-TG* triglycerides in very small VLDL, *S-LDL-TG* triglycerides in small LDL, *PUFA/MUFA* ratio of polyunsaturated fatty acids to monounsaturated fatty acids

The combination of the twelve metabolites allowed superior discrimination between COV-L and COV-M and led to a curve with significantly better performance, with AUC values ranging from 0.91 to 0.98 and a coefficient of variation prediction ranging from 0.83 to 0.92 (Table 1).

#### Biomarkers to predict severe COVID‑19 patients who will recover vs. die

The same strategy was used to develop a prognostic biomarker set to predict whether severe COVID‑19 patients will recover or die.

The top sixteen metabolites with the highest AUC values included creatinine, cholesteryl esters in small high-density lipoprotein (HDL) (S-HDL-CE), cholesterol in small HDL (S-HDL-C), the concentration of small HDL particles (S-HDL-P), the concentration of HDL particles (HDL-P), cholesteryl esters in HDL (HDL-CE), HDL cholesterol (HDL-C), the total concentration of lipoprotein particles (Total-P), cholesteryl esters in medium HDL (M-HDL-CE), cholesterol in medium HDL (M-HDL-C), PUFA/MUFA, total lipids in small HDL (S-HDL-L), unsaturation, phospholipids in small HDL (S-HDL-PL), ApoA1 and free cholesterol in medium HDL (M-HDL-FC) (Table 2). The prediction models obtained using four machine learning algorithms (random forests, linear support vector machine, PLS-DA and logistic regression) and these sixteen metabolites achieved good performance in distinguishing “recovery” from “death” among patients with severe COVID-19 (Table 2).

**Table 2:** Set of biomarkers based on 16 metabolites and prediction models based on these metabolites to distinguish patients with severe COVID‑19 who will recover or die

Area under the curve, p value, and log2 fold change for a set of biomarkers comprising 16 metabolites			
Metabolites	AUC	P value	FC
Creatinine	0.86	< 0.001	1.2358
S-HDL-CE	0.81	< 0.001	− 0.49038
S-HDL-C	0.78	< 0.001	− 0.40325
S-HDL-P	0.79	< 0.001	− 0.37039
HDL-P	0.77	< 0.001	− 0.37152
HDL-CE	0.75	< 0.001	− 0.44343
HDL-C	0.75	< 0.001	− 0.39332
Total-P	0.75	< 0.001	− 0.32648
M-HDL-CE	0.75	< 0.001	− 0.56306
M-HDL-C	0.74	< 0.001	− 0.5586
PUFA/MUFA	0.74	< 0.001	− 0.29763
S-HDL-L	0.74	< 0.001	− 0.28947
Unsaturation	0.74	< 0.001	− 0.12494
S-HDL-PL	0.74	< 0.001	− 0.28826
ApoA1	0.73	< 0.001	− 0.26444
M-HDL-FC	0.73	< 0.001	− 0.53635

Prediction models based on a set of 16 metabolites			
Algorithm	AUC	Coefficient of variation prediction	P value
PLS-DA	0.85 (0.71–0.96)	0.77	< 0.001
Linear support vector	0.87 (0.76–0.96)	0.79	0.004
Logistic regression	0.7 (0.56–0.82)	0.68	0.006
Random forest	0.84 (0.74–0.92)	0.77	< 0.001

Cholesteryl esters in small high-density lipoprotein (HDL) (S-HDL-CE), cholesterol in small HDL (S-HDL-C), concentration of small HDL particles (S-HDL-P), concentration of HDL particles (HDL-P), cholesteryl esters in HDL (HDL-CE), HDL cholesterol (HDL-C), total concentration of lipoprotein particles (Total-P), cholesteryl esters in medium HDL (M-HDL-CE), cholesterol in medium HDL (M-HDL-C), total lipids in small HDL (S-HDL-L), unsaturation, phospholipids in small HDL (S-HDL-PL), ApoA1 (Apolipoprotein A1), free cholesterol in medium HDL (M-HDL-FC)

These results indicate that fluid balance, small/medium high-density lipoprotein, fatty acid and apolipoprotein markers might serve as potential biomarkers to risk stratify patients with severe COVID-19.

### Evaluation study of hospitalized patients with severe COVID-19

A longitudinal sample of 20 severe patients with divergent outcomes (10 deceased patients and 10 discharged patients) was used to evaluate the potential prognostic value of the two biomarker sets in stratifying the risk of death from COVID-19. Initial analysis of prediction models based on two sets of biomarkers across longitudinal data from two patient groups did not yield a clear classification. This lack of a clear classification is most likely due to the irregular interval between samples, the different times of day samples were acquired, and different courses of disease progression in each patient. However, when focusing exclusively on the difference between the first and last samples of each patient, some compounds showed significant directional changes.

**Fig. 4:**
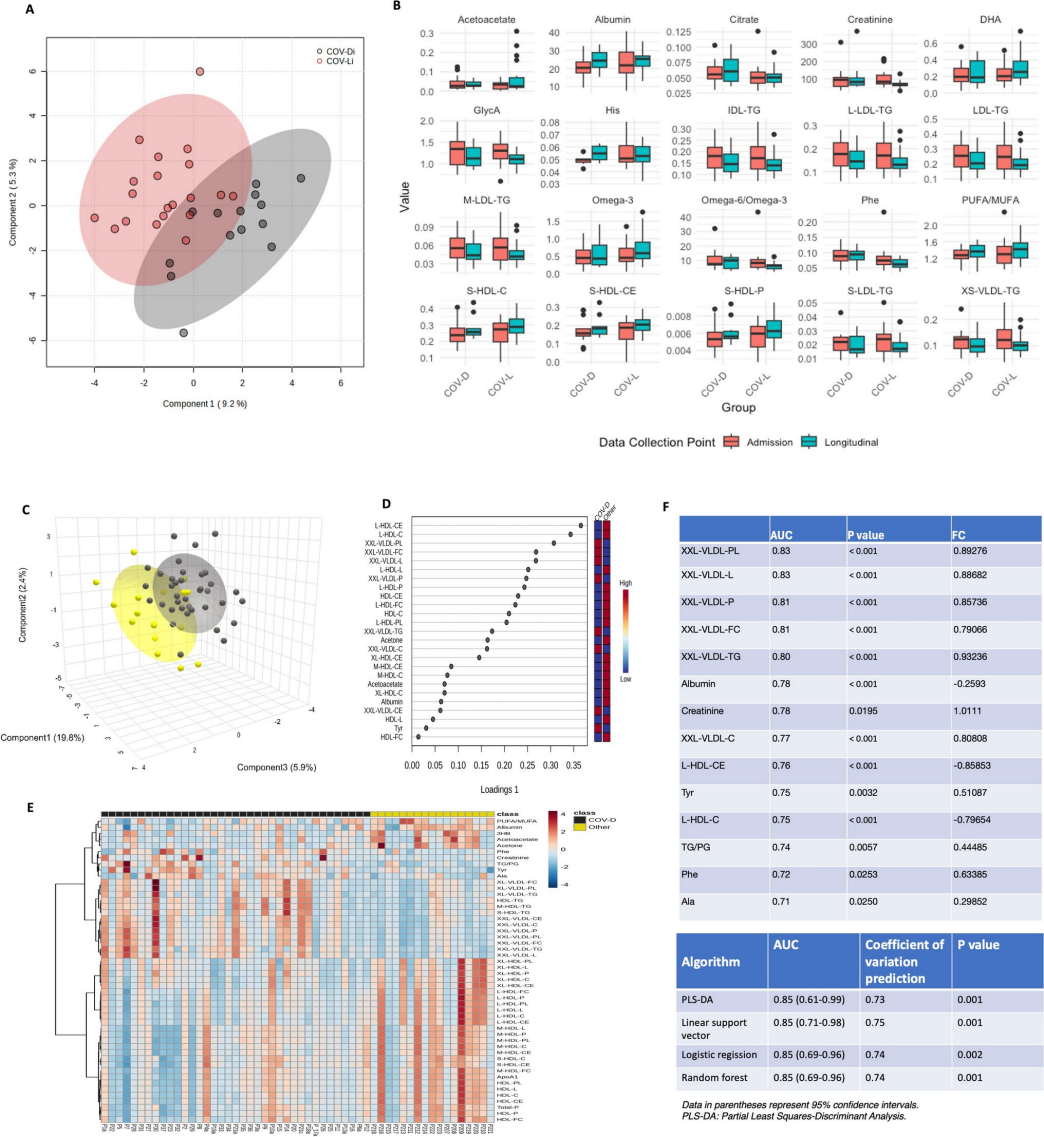
Follow-up study of patients in the hospitalized severe COVID-19 cohort and the models to distinguish patients with fatal outcomes due to severe COVID-19 vs. other diseases. **A** sPLS score plot separating the two groups of patients at the end of treatment: COV-Di (black) vs. COV-Li (red). **B** Biochemical shifts in twenty metabolites in two groups of patients, namely, severe COVID‑19 patients who will recover vs. severe COVID‑19 patients who will die. Data were obtained at two time points: at admission and at the end of treatment. **C** sPLS score plot of COV-D (black) vs. patients who died from other diseases (Other) (orange). **D** Loading plot of COV-D vs. Other. **E** Heatmap of 50 selected metabolites with a false discovery rate (FDR) < 0.05. **F** Set of biomarkers based on 15 metabolites and prediction models based on these metabolites to distinguish patients with fatal outcomes due to severe COVID-19 vs. other diseases

The first biomarker set included the twenty variables with the most significant values identified by RF to classify and validate different stages of COVID-19 (Fig. 3A). Acetoacetate, DHA and omega-3 levels were significantly increased in patients who subsequently recovered, but the levels remained unchanged in patients who died. In addition, the levels of five other metabolites, namely, albumin, PUFA/MUFA, S-HDL-C, S-HDL-CE, and S-HDL-P, were increased at the end of treatment compared to the beginning of treatment in both COV-D and COV-L groups. However, higher values were observed in discharged patients than in patients who died. In contrast, the levels of histidine and citrate were significantly increased in patients who subsequently died, but the levels remained unchanged in patients who were alive. The other ten metabolites, including creatinine, GlycA, IDL-TG, L-LDL-TG, LDL-TG, M-LDL-TG, omega-6/omega-3, phenylalanine, S-LDL-TG and XS-VLDL-TG, decreased in both groups; however lower values were observed in discharged patients (Fig. 4B). When sPLS-DA analysis was applied, the model separated the two groups of patients at the end of treatment (Fig. 4A), with an overall cross-validated accuracy of 72% (data not shown).

The second biomarker set includes fifteen metabolites that were selected to predict which patients with severe COVID‑19 will recover vs. die. The results indicated that the intensities of most of these metabolites (except creatinine) were greater at the time of recovery or death than at the time of hospitalization. However, in the group discharged from the hospital, the levels of those metabolites that were elevated seemed to return to “normal” values at the time of follow-up. In the case of creatinine, the levels remained unchanged in patients who died but were significantly decreased in patients who subsequently recovered (Fig. 4B).

The comparison of significantly altered metabolites levels from two biomarker sets in the hospitalized severe disease groups between the time of admission and at the end of treatment demonstrated that the range of severity-associated biomarkers was consistently broad. These biomarkers could potentially serve as prognostic indicators of the risk of death from COVID-19 in severely ill patients.

### Models to distinguish patients with fatal outcomes due to severe COVID-19 vs. other diseases

Focusing on any difference in the metabolome between patients with fatal outcomes from severe COVID-19 and those with fatal outcomes from other diseases, an sPLS-DA model was built. The sPLS score plot showed separation, with an overall cross-validated accuracy of 76% (Fig. 4C). COVID-19 patients with fatal outcomes presented lower levels of cholesteryl esters in large HDL (L-HDL-CE), cholesterol in large HDL (L-HDC-C), total lipids in large HDL (L-HDL-L), concentrations of large HDL particles (L-HDL-P), HDL-CE, free cholesterol in large HDL (L-HDL-FC), HDL cholesterol (HDL-C), phospholipids in large HDL (L-HDL-PL), acetone, cholesteryl esters in very large HDL (XL-HDL-CE), M-HDL-CE, M-HDL-C, acetoacetate, free cholesterol in large HDL (L-HDL-FC), albumin, total lipids in HDL (HDL-L) and free cholesterol in HDL (HDL-FC) as well as higher levels of phospholipids in chylomicrons and extremely large VLDL (XXL-VLDL-PL), free cholesterol in chylomicrons and extremely large VLDL (XXL-VLDL-FC), total lipids in chylomicrons and extremely large VLDL (XXL-VLDL-L), concentrations of chylomicrons and extremely large VLDL particles (XXL-VLDL-P), triglycerides in chylomicrons and extremely large VLDL (XXL-VLDL-TG), cholesterol in chylomicrons and extremely large VLDL (XXL-VLDL-C), cholesteryl esters in chylomicrons and extremely large VLDL (XXL-VLDL-CE) and tyrosine (Tyr) compared to patients who died from other diseases (Fig. 4D). Some of the metabolites, such as acetoacetate and albumin, also differed among COVID-19 patients (Fig. 3A, and B).

Similar results for those altered metabolites obtained via sPLS analysis were also found as demonstrated in the heatmap (Fig. 4E).

Finally, the same procedure was applied to identify a prognostic biomarker set capable of distinguishing whether fatal patients belonged to the COVID-19 group or the group with other illnesses. This biomarker set comprised fifteen metabolites, including XXL-VLDL-PL, XXL-VLDL-L, XXL-VLDL-P, XXL-VLDL-FC, XXL-VLDL-TG, albumin, creatinine, XXL-VLDL-C, L-HDL-CE, tyrosine (Tyr), L-HDL-C, TG/PG, phenylalanine (Phe), and alanine (Ala). The results showed that the intensities of most of these metabolites—except albumin, L-HDL-CE, and L-HDL-C—were higher in the COVID-19 group compared to the Other-D group. Detailed information is summarized in (Fig. 4F).

The combination of these fifteen metabolites enabled a clear distinction between the COV-D and Other-D groups, resulting in a predictive model with strong performance: an AUC value of 0.85 and a prediction coefficient of variation ranging from 0.73 to 0.75 (Fig. 4F).

## Discussion

In this study, we provide a comprehensive view of the metabolomic changes in serum among COVID-19 patients at all stages, patients with mild to severe COVID-19 with or without recovery, and patients with fatal outcomes with and without COVID-19. Our results revealed that distinct alterations in the serum metabolome were capable of identifying patients with COVID-19 in terms of either severity (mild/severe) or outcome (dead/survived) and identifying patients with fatal outcomes (COVID-19/other disease). The random forest model and sPLS model clearly revealed differences in both the metabolomic profiles of COVID-19 patients with mild or moderate disease vs. severe disease as well as between patients with severe COVID-19 who recovered (COV-L) vs. died (COV-D) (Figs. 2C, 3E and F). We achieved an overall accuracy of 80% in both the training and validation sets (Fig. 3C and D). The results from our study align well with many other studies on COVID-19 disease using metabolomics; these results confirm that the high-throughput metabolomics approach could be used to predict disease development (Barberis et al., 2020; Blasco et al., 2020; Dierckx et al., 2020; Shen et al., 2020; Song et al., 2020; Thomas et al., 2020).

In fact, the metabolome of COVID-19 patients with mild/moderate disease was very close to that of the non-COVID-19 control group, and these two groups could not be distinguished. Unlike most early studies, which applied metabolomics to compare COVID-19 patients with healthy controls (Barberis et al., 2020; Blasco et al., 2020; Dierckx et al., 2020), the differences might be due to the non-COVID-19 control in this study, which included people who had symptoms similar to those of COVID-19 but were negative according to real-time PCR.

More importantly, using ROC curve analysis and four machine learning algorithms (random forest, linear support vector machine, PLS-DA, and logistic regression), we identified two biomarker sets with relevant biological functions that predict subsequent disease severity and patient outcome. Notably, those metabolites from the two biomarker sets not only showed a gradual metabolic transition from the mild/moderate phase to the severe phase of the COVID-19 stage but could also be used to accurately differentiate severe patients who will recover from those who will eventually die (Figs. 2B, 4).

The significant gradient of COVID severity was evident across groupings based on amino acids, inflammation, fluid balance, ketone bodies, glycolysis-related metabolites, and lipids. Low levels of albumin, histidine, PUFA/MUFA, S-HDL-C, S-HDL-CE, and S-HDL-P and high levels of, creatinine, GlycA, IDL-TG, L-LDL-TG, LDL-TG, M-LDL-TG, phenylalanine, S-HDL-TG and XS-VLDL-TG were associated with increased infection severity.

The variation in the lipid profile at different stages of COVID-19 and its association with disease severity are not surprising. Low HDL-C concentrations and high triglyceride levels have previously been linked to worse outcomes of sepsis, specifically respiratory viral sepsis (Tanaka et al., 2020). With respect to SARS-CoV-2 infection, low HDL-C and triglyceride (TG) levels are associated with increasing infection severity (Abu-Farha et al., 2020; Wei et al., 2020), and a role for these lipids in immune mechanisms has been suggested (Harris et al., 2000). Our results confirm reports that low HDL-C levels at admission are associated with greater severity and worse clinical outcomes in patients with COVID-19 (Dierckx et al., 2020; Parra et al., 2023; Shen et al., 2020). Shen et al. reported that more than one hundred lipids, including glycerophospholipids, sphingolipids, and fatty acids, were downregulated in patients with severe COVID-19, probably because of damage to the liver, which is also reflected in abnormalities in the levels of bilirubin and bile acids.

On the other hand, the variation in the lipid profile due to COVID-19 is associated with disease severity, probably because infection interferes with several steps of lipid metabolism. This process may result from reduced cholesterol synthesis and absorption, decreased triglyceride-rich lipoprotein clearance or reduced apolipoprotein (apo) A1 synthesis. Consequently, these processes generate cytokines, inflammatory mediators, modified lipids and intermediate lipid classes (Danlos et al., 2021).

Albumin, a protein that exerts important homeostatic effects, including maintaining osmotic colloid pressure, intravascular transport of molecules, and lipid metabolism, is classically considered a biomarker of malnutrition and poor health status (Nazha, 2015). The evidence for the use of the serum albumin concentration as a marker of severity is not new. Low serum albumin concentrations are associated with the severity of chronic inflammatory diseases (Din et al., 2020), liver disease (Spinella et al., 2015), surgical stress (Hübner et al., 2016), and acute diseases (Sahin et al., 2018). Furthermore, in past SARS epidemics, hypoalbuminemia has been shown to be related to disease severity and increased hospital mortality (Chan et al., 2007; Leong et al., 2006). In addition, a systematic review and meta-analysis investigated the associations between the serum albumin concentration and COVID-19 disease severity and adverse outcomes, demonstrating that lower serum albumin concentrations are significantly associated with disease severity and adverse outcomes in COVID-19 patients (Paliogiannis et al., 2021). Albumin concentrations were also inversely correlated with inflammation markers. Our analysis revealed markedly lower levels of albumin and significantly higher levels of GlycA in the severe COVID-19 patient group than in the moderate COVID-19 patient group. Notably, our analyses also revealed low albumin levels in patients who died from other diseases. Thus, this study reinforces the findings that the serum ALB concentration is a marker of disease severity in patients with COVID-19 infection as well as in those with other infectious diseases.

According to Wannemacher et al., inflammation and infection often lead to increased levels of various serum amino acids, especially serum phenylalanine (Wannemacher, 1977). This occurs due to accelerated release from skeletal muscle to meet the rates of glucose turnover and oxidation in the infected host (Wannemacher et al., 1976). Recently, Lima et al. (2024) demonstrated markedly higher phenylalanine levels among subjects with moderate to severe disease. This is probably because inflammatory cytokines promote muscle breakdown by releasing phenylalanine for gluconeogenesis to meet the metabolic demand during infection. Thus, higher phenylalanine levels are associated with the catabolic state. Our results agree with the significant association between higher phenylalanine and GlycA levels and COVID-19 severity (Dierckx et al., 2020). This association is also consistent with reports of a low occurrence of severe COVID-19 among patients with phenylketonuria, which has previously been attributed to the lower rates of vitamin D deficiency in these patients as a result of protein substitutes in their diet (Rocha et al., 2020).

This study is the first to compare metabolomic differences between patients who died from COVID-19 and those who died from other, non-COVID-19-related illnesses. Notably, both groups were composed of elderly individuals with relatively homogeneous characteristics. The median age of COVID-19 patients who died in our study was 85 years, which is comparable to those reported in studies from the UK (median age 80 years) (Martins-Filho et al., 2020) and Denmark (median age 81 years) (Hodges et al., 2020).

The initial gland view with the heatmap indicates that approximately 50% of patients in the Other-D cohort exhibit lipid concentration profiles similar to those observed in the COV-D cohort. As is well established, lipids play critical roles in cellular function—they participate in signal transduction, preserve the structural integrity of cell membranes, and regulate energy metabolism. Lipid metabolism also underpins essential processes for cellular homeostasis, including membrane synthesis and the storage and mobilization of energy via triglycerides (Yoon et al., 2021).

The observed similarities in lipid concentrations between the two groups may be attributed to: (1) age-related immune dysregulation (inflammaging), which disrupts glucose, lipid, and amino acid metabolism; and (2) dysregulated lipid metabolism, which promotes systemic inflammation and contributes to organ damage in both cohorts.

However, when we focused more specifically on the fifteen metabolites most critical for distinguishing metabolic differences between the two groups, our analysis revealed elevated levels of VLDL and VLDL-associated cholesterol in the COV-D patient group compared to the Other-D group. These findings suggest a disrupted energy balance and potential hepatic damage in COVID-19 patients.

This supports previous reports indicating that elevated triglycerides and cholesterol in VLDL contribute to the progression of severe COVID-19 (Schmelter et al., 2021). Furthermore, a recent systematic study demonstrated that the formation of syncytia—a key process in viral entry mediated by the ACE2 receptor—requires cholesterol (Sanders et al. 2021). From this perspective, the cholesterol and cholesterol esters within specific VLDL particles may facilitate membrane fusion events essential for viral infection. Collectively, these findings highlight a distinct metabolic signature associated with COVID-19.

## Limitaions

This study has several limitations that should be taken into account when interpreting the findings. The primary limitation is that practical constraints, such as limited sample availability, resulted in cohort biases, particularly in the age and sex distribution across different groups. For example, a higher proportion of male participants was observed in the hospitalized cohorts—most notably in the COV-D group (78%). Additionally, the median age was significantly higher in the hospitalized and deceased groups (COV-D: 85.4 ± 8.4 years; Other-D: 84 ± 8.7 years), which may limit the statistical power to draw definitive conclusions about group differences. Future studies with larger, more demographically balanced cohorts are necessary to better understand age- and sex-specific variations in COVID-19 outcomes. Furthermore, various confounding factors—such as nutritional status, existing comorbidities, and insufficient documentation of medications—may have influenced biomarker levels.

## Conclusions

Our data demonstrate that distinct alterations in the serum metabolome can effectively identify patients with COVID-19 based on severity (mild/severe) or outcome (death/survival). Two biomarker sets associated with relevant biological functions provide strong predictive value for subsequent disease severity and patient outcome. The range of these severity-associated biomarkers was broad and included inflammatory markers (glycoprotein acetylation), amino acids (histidine, phenylalanine), fluid balance (albumin, creatinine), ketone bodies (acetoacetate), glycolysis-related metabolites (glucose), lipoprotein particles (only HDL), cholesterol levels (small/medium HDL and very small/small VLDL), triglyceride levels (only IDL and LDL), and fatty acid levels (polyunsaturated fatty acid, monounsaturated fatty acid). Our data suggests the potential benefits of broader testing of these metabolites in newly diagnosed patients to predict which COVID-19 patients are likely to progress to severe disease versus experience more severe symptoms to minimize mortality.

## Supplementary Information

Below is the link to the electronic supplementary material.

Supplementary file1 (DOCX 222 KB)

## Data Availability

No datasets were generated or analysed during the current study.
