# Supplementary material for: Metabolic signature of COVID-19 progression: potential prognostic markers for severity and outcome
Source: Metabolomics. 2025 May 21;21(3):70. doi: 10.1007/s11306-025-02264-w (PMC12095333; doi:10.1007/s11306-025-02264-w)
Supplement: Supplementary file 1 — Supplementary file1 (DOCX 222 KB) [file 11306_2025_2264_MOESM1_ESM.docx]

**SUPPEMENTARY TABLES**

Table S0: List of 172 metabolites identified by serum metabolomics

| **No** | **Metabolites** | **Short name** | | **Group** | |
| --- | --- | --- | --- | --- | --- |
| 1 | Total cholesterol | Total_C | Cholesterol | |  |
| 2 | Total cholesterol minus HDL-C | non_HDL_C | Cholesterol | |  |
| 3 | Remnant cholesterol (non-HDL, non-LDL -cholesterol) | Remnant_C | Cholesterol | |  |
| 4 | VLDL cholesterol | VLDL_C | Cholesterol | |  |
| 5 | Clinical LDL cholesterol | Clinical_LDL_C | Cholesterol | |  |
| 6 | LDL cholesterol | LDL_C | Cholesterol | |  |
| 7 | HDL cholesterol | HDL_C | Cholesterol | |  |
| 8 | Total triglycerides | Total_TG | Triglycerides | |  |
| 9 | Triglycerides in VLDL | VLDL_TG | Triglycerides | |  |
| 10 | Triglycerides in LDL | LDL_TG | Triglycerides | |  |
| 11 | Triglycerides in HDL | HDL_TG | Triglycerides | |  |
| 12 | Total phospholipids in lipoprotein particles | Total_PL | Phospholipids | |  |
| 13 | Phospholipids in VLDL | VLDL_PL | Phospholipids | |  |
| 14 | Phospholipids in LDL | LDL_PL | Phospholipids | |  |
| 15 | Phospholipids in HDL | HDL_PL | Phospholipids | |  |
| 16 | Total esterified cholesterol | Total_CE | Cholesteryl esters | |  |
| 17 | Cholesteryl esters in VLDL | VLDL_CE | Cholesteryl esters | |  |
| 18 | Cholesteryl esters in LDL | LDL_CE | Cholesteryl esters | |  |
| 19 | Cholesteryl esters in HDL | HDL_CE | Cholesteryl esters | |  |
| 20 | Total free cholesterol | Total_FC | Free cholesterol | |  |
| 21 | Free cholesterol in VLDL | VLDL_FC | Free cholesterol | |  |
| 22 | Free cholesterol in LDL | LDL_FC | Free cholesterol | |  |
| 23 | Free cholesterol in HDL | HDL_FC | Free cholesterol | |  |
| 24 | Total lipids in lipoprotein particles | Total_L | Total lipids | |  |
| 25 | Total lipids in VLDL | VLDL_L | Total lipids | |  |
| 26 | Total lipids in LDL | LDL_L | Total lipids | |  |
| 27 | Total lipids in HDL | HDL_L | Total lipids | |  |
| 28 | Total concentration of lipoprotein particles | Total_P | Lipoprotein particle concentrations | |  |
| 29 | Concentration of VLDL particles | VLDL_P | Lipoprotein particle concentrations | |  |
| 30 | Concentration of LDL particles | LDL_P | Lipoprotein particle concentrations | |  |
| 31 | Concentration of HDL particles | HDL_P | Lipoprotein particle concentrations | |  |
| 32 | Average diameter for VLDL particles | VLDL_size | Lipoprotein particle sizes | |  |
| 33 | Average diameter for LDL particles | LDL_size | Lipoprotein particle sizes | |  |
| 34 | Average diameter for HDL particles | HDL_size | Lipoprotein particle sizes | |  |
| 35 | Phosphoglycerides | Phosphoglyc | Other lipids | |  |
| 36 | Total cholines | Cholines | Other lipids | |  |
| 37 | Phosphatidylcholines | Phosphatidylc | Other lipids | |  |
| 38 | Sphingomyelins | Sphingomyelins | Other lipids | |  |
| 39 | Apolipoprotein B | ApoB | Apolipoproteins | |  |
| 40 | Apolipoprotein A1 | ApoA1 | Apolipoproteins | |  |
| 41 | Ratio of apolipoprotein B to apolipoprotein A1 | ApoB_by_ApoA1 | Apolipoproteins | |  |
| 42 | Total fatty acids | Total_FA | Fatty acids | |  |
| 43 | Degree of unsaturation | Unsaturation | Fatty acids | |  |
| 44 | Omega-3 fatty acids | Omega_3 | Fatty acids | |  |
| 45 | Omega-6 fatty acids | Omega_6 | Fatty acids | |  |
| 46 | Polyunsaturated fatty acids | PUFA | Fatty acids | |  |
| 47 | Monounsaturated fatty acids | MUFA | Fatty acids | |  |
| 48 | Saturated fatty acids | SFA | Fatty acids | |  |
| 49 | Linoleic acid | LA | Fatty acids | |  |
| 50 | Docosahexaenoic acid | DHA | Fatty acids | |  |
| 51 | Ratio of polyunsaturated fatty acids to monounsaturated fatty acids | PUFA_by_MUFA | Fatty acids | |  |
| 52 | Ratio of omega-6 fatty acids to omega-3 fatty acids | Omega_6_by_Omega_3 | Fatty acids | |  |
| 53 | Alanine | Ala | Amino acids | |  |
| 54 | Glutamine | Gln | Amino acids | |  |
| 55 | Glycine | Gly | Amino acids | |  |
| 56 | Histidine | His | Amino acids | |  |
| 57 | Total concentration of branched-chain amino acids (leucine + isoleucine + valine) | Total_BCAA | Amino acids | |  |
| 58 | Isoleucine | Ile | Amino acids | |  |
| 59 | Leucine | Leu | Amino acids | |  |
| 60 | Valine | Val | Amino acids | |  |
| 61 | Phenylalanine | Phe | Amino acids | |  |
| 62 | Tyrosine | Tyr | Amino acids | |  |
| 63 | Glucose | Glucose | Glycolysis related metabolites | |  |
| 64 | Lactate | Lactate | Glycolysis related metabolites | |  |
| 65 | Pyruvate | Pyruvate | Glycolysis related metabolites | |  |
| 66 | Citrate | Citrate | Glycolysis related metabolites | |  |
| 67 | Glycerol | Glycerol | Glycolysis related metabolites | |  |
| 68 | 3-Hydroxybutyrate | bOHbutyrate | Ketone bodies | |  |
| 69 | Acetate | Acetate | Ketone bodies | |  |
| 70 | Acetoacetate | Acetoacetate | Ketone bodies | |  |
| 71 | Acetone | Acetone | Ketone bodies | |  |
| 72 | Creatinine | Creatinine | Fluid balance | |  |
| 73 | Albumin | Albumin | Fluid balance | |  |
| 74 | Glycoprotein acetyls | GlycA | Inflammation | |  |
| 75 | Concentration of chylomicrons and extremely large VLDL particles | XXL_VLDL_P | Lipoprotein subclasses | |  |
| 76 | Total lipids in chylomicrons and extremely large VLDL | XXL_VLDL_L | Lipoprotein subclasses | |  |
| 77 | Phospholipids in chylomicrons and extremely large VLDL | XXL_VLDL_PL | Lipoprotein subclasses | |  |
| 78 | Cholesterol in chylomicrons and extremely large VLDL | XXL_VLDL_C | Lipoprotein subclasses | |  |
| 79 | Cholesteryl esters in chylomicrons and extremely large VLDL | XXL_VLDL_CE | Lipoprotein subclasses | |  |
| 80 | Free cholesterol in chylomicrons and extremely large VLDL | XXL_VLDL_FC | Lipoprotein subclasses | |  |
| 81 | Triglycerides in chylomicrons and extremely large VLDL | XXL_VLDL_TG | Lipoprotein subclasses | |  |
| 82 | Concentration of very large VLDL particles | XL_VLDL_P | Lipoprotein subclasses | |  |
| 83 | Total lipids in very large VLDL | XL_VLDL_L | Lipoprotein subclasses | |  |
| 84 | Phospholipids in very large VLDL | XL_VLDL_PL | Lipoprotein subclasses | |  |
| 85 | Cholesterol in very large VLDL | XL_VLDL_C | Lipoprotein subclasses | |  |
| 86 | Cholesteryl esters in very large VLDL | XL_VLDL_CE | Lipoprotein subclasses | |  |
| 87 | Free cholesterol in very large VLDL | XL_VLDL_FC | Lipoprotein subclasses | |  |
| 88 | Triglycerides in very large VLDL | XL_VLDL_TG | Lipoprotein subclasses | |  |
| 89 | Concentration of large VLDL particles | L_VLDL_P | Lipoprotein subclasses | |  |
| 90 | Total lipids in large VLDL | L_VLDL_L | Lipoprotein subclasses | |  |
| 91 | Phospholipids in large VLDL | L_VLDL_PL | Lipoprotein subclasses | |  |
| 92 | Cholesterol in large VLDL | L_VLDL_C | Lipoprotein subclasses | |  |
| 93 | Cholesteryl esters in large VLDL | L_VLDL_CE | Lipoprotein subclasses | |  |
| 94 | Free cholesterol in large VLDL | L_VLDL_FC | Lipoprotein subclasses | |  |
| 95 | Triglycerides in large VLDL | L_VLDL_TG | Lipoprotein subclasses | |  |
| 96 | Concentration of medium VLDL particles | M_VLDL_P | Lipoprotein subclasses | |  |
| 97 | Total lipids in medium VLDL | M_VLDL_L | Lipoprotein subclasses | |  |
| 98 | Phospholipids in medium VLDL | M_VLDL_PL | Lipoprotein subclasses | |  |
| 99 | Cholesterol in medium VLDL | M_VLDL_C | Lipoprotein subclasses | |  |
| 100 | Cholesteryl esters in medium VLDL | M_VLDL_CE | Lipoprotein subclasses | |  |
| 101 | Free cholesterol in medium VLDL | M_VLDL_FC | Lipoprotein subclasses | |  |
| 102 | Triglycerides in medium VLDL | M_VLDL_TG | Lipoprotein subclasses | |  |
| 103 | Concentration of small VLDL particles | S_VLDL_P | Lipoprotein subclasses | |  |
| 104 | Total lipids in small VLDL | S_VLDL_L | Lipoprotein subclasses | |  |
| 105 | Phospholipids in small VLDL | S_VLDL_PL | Lipoprotein subclasses | |  |
| 106 | Cholesterol in small VLDL | S_VLDL_C | Lipoprotein subclasses | |  |
| 107 | Cholesteryl esters in small VLDL | S_VLDL_CE | Lipoprotein subclasses | |  |
| 108 | Free cholesterol in small VLDL | S_VLDL_FC | Lipoprotein subclasses | |  |
| 109 | Triglycerides in small VLDL | S_VLDL_TG | Lipoprotein subclasses | |  |
| 110 | Concentration of very small VLDL particles | XS_VLDL_P | Lipoprotein subclasses | |  |
| 111 | Total lipids in very small VLDL | XS_VLDL_L | Lipoprotein subclasses | |  |
| 112 | Phospholipids in very small VLDL | XS_VLDL_PL | Lipoprotein subclasses | |  |
| 113 | Cholesterol in very small VLDL | XS_VLDL_C | Lipoprotein subclasses | |  |
| 114 | Cholesteryl esters in very small VLDL | XS_VLDL_CE | Lipoprotein subclasses | |  |
| 115 | Free cholesterol in very small VLDL | XS_VLDL_FC | Lipoprotein subclasses | |  |
| 116 | Triglycerides in very small VLDL | XS_VLDL_TG | Lipoprotein subclasses | |  |
| 117 | Concentration of IDL particles | IDL_P | Lipoprotein subclasses | |  |
| 118 | Total lipids in IDL | IDL_L | Lipoprotein subclasses | |  |
| 119 | Phospholipids in IDL | IDL_PL | Lipoprotein subclasses | |  |
| 120 | Cholesterol in IDL | IDL_C | Lipoprotein subclasses | |  |
| 121 | Cholesteryl esters in IDL | IDL_CE | Lipoprotein subclasses | |  |
| 122 | Free cholesterol in IDL | IDL_FC | Lipoprotein subclasses | |  |
| 123 | Triglycerides in IDL | IDL_TG | Lipoprotein subclasses | |  |
| 124 | Concentration of large LDL particles | L_LDL_P | Lipoprotein subclasses | |  |
| 125 | Total lipids in large LDL | L_LDL_L | Lipoprotein subclasses | |  |
| 126 | Phospholipids in large LDL | L_LDL_PL | Lipoprotein subclasses | |  |
| 127 | Cholesterol in large LDL | L_LDL_C | Lipoprotein subclasses | |  |
| 128 | Cholesteryl esters in large LDL | L_LDL_CE | Lipoprotein subclasses | |  |
| 129 | Free cholesterol in large LDL | L_LDL_FC | Lipoprotein subclasses | |  |
| 130 | Triglycerides in large LDL | L_LDL_TG | Lipoprotein subclasses | |  |
| 131 | Concentration of medium LDL particles | M_LDL_P | Lipoprotein subclasses | |  |
| 132 | Total lipids in medium LDL | M_LDL_L | Lipoprotein subclasses | |  |
| 133 | Phospholipids in medium LDL | M_LDL_PL | Lipoprotein subclasses | |  |
| 134 | Cholesterol in medium LDL | M_LDL_C | Lipoprotein subclasses | |  |
| 135 | Cholesteryl esters in medium LDL | M_LDL_CE | Lipoprotein subclasses | |  |
| 136 | Free cholesterol in medium LDL | M_LDL_FC | Lipoprotein subclasses | |  |
| 137 | Triglycerides in medium LDL | M_LDL_TG | Lipoprotein subclasses | |  |
| 138 | Concentration of small LDL particles | S_LDL_P | Lipoprotein subclasses | |  |
| 139 | Total lipids in small LDL | S_LDL_L | Lipoprotein subclasses | |  |
| 140 | Phospholipids in small LDL | S_LDL_PL | Lipoprotein subclasses | |  |
| 141 | Cholesterol in small LDL | S_LDL_C | Lipoprotein subclasses | |  |
| 142 | Cholesteryl esters in small LDL | S_LDL_CE | Lipoprotein subclasses | |  |
| 143 | Free cholesterol in small LDL | S_LDL_FC | Lipoprotein subclasses | |  |
| 144 | Triglycerides in small LDL | S_LDL_TG | Lipoprotein subclasses | |  |
| 145 | Concentration of very large HDL particles | XL_HDL_P | Lipoprotein subclasses | |  |
| 146 | Total lipids in very large HDL | XL_HDL_L | Lipoprotein subclasses | |  |
| 147 | Phospholipids in very large HDL | XL_HDL_PL | Lipoprotein subclasses | |  |
| 148 | Cholesterol in very large HDL | XL_HDL_C | Lipoprotein subclasses | |  |
| 149 | Cholesteryl esters in very large HDL | XL_HDL_CE | Lipoprotein subclasses | |  |
| 150 | Free cholesterol in very large HDL | XL_HDL_FC | Lipoprotein subclasses | |  |
| 151 | Triglycerides in very large HDL | XL_HDL_TG | Lipoprotein subclasses | |  |
| 152 | Concentration of large HDL particles | L_HDL_P | Lipoprotein subclasses | |  |
| 153 | Total lipids in large HDL | L_HDL_L | Lipoprotein subclasses | |  |
| 154 | Phospholipids in large HDL | L_HDL_PL | Lipoprotein subclasses | |  |
| 155 | Cholesterol in large HDL | L_HDL_C | Lipoprotein subclasses | |  |
| 156 | Cholesteryl esters in large HDL | L_HDL_CE | Lipoprotein subclasses | |  |
| 157 | Free cholesterol in large HDL | L_HDL_FC | Lipoprotein subclasses | |  |
| 158 | Triglycerides in large HDL | L_HDL_TG | Lipoprotein subclasses | |  |
| 159 | Concentration of medium HDL particles | M_HDL_P | Lipoprotein subclasses | |  |
| 160 | Total lipids in medium HDL | M_HDL_L | Lipoprotein subclasses | |  |
| 161 | Phospholipids in medium HDL | M_HDL_PL | Lipoprotein subclasses | |  |
| 162 | Cholesterol in medium HDL | M_HDL_C | Lipoprotein subclasses | |  |
| 163 | Cholesteryl esters in medium HDL | M_HDL_CE | Lipoprotein subclasses | |  |
| 164 | Free cholesterol in medium HDL | M_HDL_FC | Lipoprotein subclasses | |  |
| 165 | Triglycerides in medium HDL | M_HDL_TG | Lipoprotein subclasses | |  |
| 166 | Concentration of small HDL particles | S_HDL_P | Lipoprotein subclasses | |  |
| 167 | Total lipids in small HDL | S_HDL_L | Lipoprotein subclasses | |  |
| 168 | Phospholipids in small HDL | S_HDL_PL | Lipoprotein subclasses | |  |
| 169 | Cholesterol in small HDL | S_HDL_C | Lipoprotein subclasses | |  |
| 170 | Cholesteryl esters in small HDL | S_HDL_CE | Lipoprotein subclasses | |  |
| 171 | Free cholesterol in small HDL | S_HDL_FC | Lipoprotein subclasses | |  |
| 172 | Triglycerides in small HDL | S_HDL_TG | Lipoprotein subclasses | |  |

Table S1. Baseline characteristics of non-COVID-19 patients and COVID-19 patients at admission.


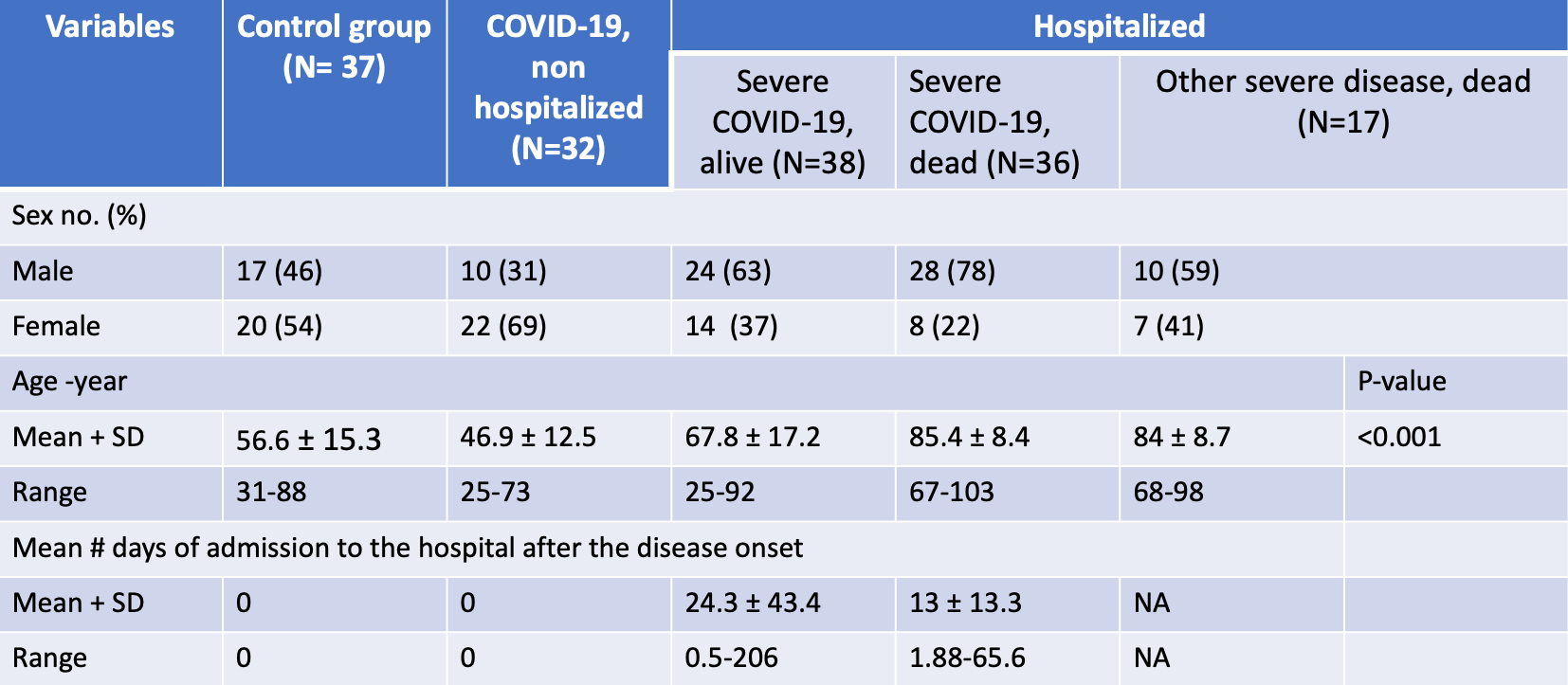


Table S2: 118 metabolites that are significantly different between at least one pair of groups revealed by the ANOVA with Fisher’s LDL test.

| **Metabolites** | **f.value** | **p.value** | **-log10(p)** | **FDR** | **Fisher's LSD** |
| --- | --- | --- | --- | --- | --- |
| **Albumin** | 81.02 | < 0.001 | 40.185 | 1.1233e-38 | COV-L - COV-D; COV-M - COV-D; HEA - COV-D; Other - COV-D; COV-M - COV-L; HEA - COV-L; COV-M - Other; HEA - Other |
| **GlycA** | 30.706 | < 0.001 | 19.243 | 4.9144e-18 | COV-D - COV-L; COV-D - COV-M; COV-D - HEA; COV-L - COV-M; COV-L - HEA; Other - COV-M; Other - HEA |
| **S-HDL-CE** | 28.914 | < 0.001 | 18.28 | 3.0091e-17 | COV-L - COV-D; COV-M - COV-D; HEA - COV-D; Other - COV-D; COV-M - COV-L; HEA - COV-L; COV-M - Other; HEA - Other |
| **M-HDL-CE** | 25.577 | < 0.001 | 16.43 | 1.4141e-15 | COV-L - COV-D; COV-M - COV-D; HEA - COV-D; Other - COV-D; COV-M - COV-L; HEA - COV-L; COV-M - Other; HEA - Other |
| **PUFA/MUFA** | 25.498 | < 0.001 | 16.386 | 1.4141e-15 | COV-L - COV-D; COV-M - COV-D; HEA - COV-D; COV-M - COV-L; HEA - COV-L; COV-M - Other; HEA - Other |
| **S-HDL-C** | 24.775 | < 0.001 | 15.975 | 3.0387e-15 | COV-L - COV-D; COV-M - COV-D; HEA - COV-D; Other - COV-D; COV-M - COV-L; HEA - COV-L; HEA - COV-M; COV-M - Other; HEA - Other |
| **HDL-P** | 24.492 | < 0.001 | 15.813 | 3.3389e-15 | COV-L - COV-D; COV-M - COV-D; HEA - COV-D; Other - COV-D; COV-M - COV-L; HEA - COV-L; COV-M - Other; HEA - Other |
| **M-HDL-C** | 24.485 | < 0.001 | 15.809 | 3.3389e-15 | COV-L - COV-D; COV-M - COV-D; HEA - COV-D; Other - COV-D; COV-M - COV-L; HEA - COV-L; COV-M - Other; HEA - Other |
| **S-HDL-P** | 24.2 | < 0.001 | 15.645 | 4.3291e-15 | COV-L - COV-D; COV-M - COV-D; HEA - COV-D; Other - COV-D; COV-M - COV-L; HEA - COV-L; HEA - COV-M; COV-M - Other; HEA - Other |
| **M-HDL-P** | 23.086 | < 0.001 | 15 | 1,721E-11 | COV-L - COV-D; COV-M - COV-D; HEA - COV-D; Other - COV-D; COV-M - COV-L; HEA - COV-L; COV-M - Other; HEA - Other |
| **HDL-CE** | 21.86 | < 0.001 | 14.278 | 8.2388e-14 | COV-L - COV-D; COV-M - COV-D; HEA - COV-D; Other - COV-D; COV-M - COV-L; HEA - COV-L; HEA - Other |
| **S-HDL-PL** | 21.645 | < 0.001 | 14.151 | 1.0122e-13 | COV-L - COV-D; COV-M - COV-D; HEA - COV-D; COV-M - COV-L; HEA - COV-L; HEA - COV-M; COV-M - Other; HEA - Other |
| **ApoA1** | 21.268 | < 0.001 | 13.926 | 1.5671e-13 | COV-L - COV-D; COV-M - COV-D; HEA - COV-D; Other - COV-D; COV-M - COV-L; HEA - COV-L; COV-M - Other; HEA - Other |
| **M-HDL-L** | 20.914 | < 0.001 | 13.715 | 2.3708e-13 | COV-L - COV-D; COV-M - COV-D; HEA - COV-D; Other - COV-D; COV-M - COV-L; HEA - COV-L; COV-M - Other; HEA - Other |
| **Total-P** | 20.864 | < 0.001 | 13.684 | 2.3724e-13 | COV-L - COV-D; COV-M - COV-D; HEA - COV-D; Other - COV-D; COV-M - COV-L; HEA - COV-L; COV-M - Other; HEA - Other |
| **S-HDL-L** | 20.151 | < 0.001 | 13.255 | 5.9828e-13 | COV-L - COV-D; COV-M - COV-D; HEA - COV-D; COV-M - COV-L; HEA - COV-L; HEA - COV-M; COV-M - Other; HEA - Other |
| **HDL-C** | 19.412 | < 0.001 | 12.804 | 1.5885e-12 | COV-L - COV-D; COV-M - COV-D; HEA - COV-D; Other - COV-D; COV-M - COV-L; HEA - COV-L; HEA - Other |
| **M-HDL-PL** | 19.008 | < 0.001 | 12.557 | 2,653E-09 | COV-L - COV-D; COV-M - COV-D; HEA - COV-D; Other - COV-D; COV-M - COV-L; HEA - COV-L; COV-M - Other; HEA - Other |
| **TG/PG** | 18.078 | < 0.001 | 11.981 | 9.4558e-12 | COV-D - COV-L; COV-D - COV-M; COV-D - HEA; COV-D - Other; COV-L - COV-M; COV-L - HEA; Other - COV-M; Other - HEA |
| **M-HDL-FC** | 17.991 | < 0.001 | 11.927 | 1.0184e-11 | COV-L - COV-D; COV-M - COV-D; HEA - COV-D; Other - COV-D; COV-M - COV-L; HEA - COV-L; HEA - Other |
| **Creatinine** | 17.214 | < 0.001 | 11.44 | 2.9756e-11 | COV-D - COV-L; COV-D - COV-M; COV-D - HEA; COV-D - Other |
| **HDL-L** | 16.609 | < 0.001 | 11.057 | 6.8531e-11 | COV-L - COV-D; COV-M - COV-D; HEA - COV-D; Other - COV-D; COV-M - COV-L; HEA - COV-L; HEA - Other |
| **HDL-PL** | 16.26 | < 0.001 | 10.836 | 1.0919e-10 | COV-L - COV-D; COV-M - COV-D; HEA - COV-D; Other - COV-D; COV-M - COV-L; HEA - COV-L; HEA - Other |
| **IDL-TG** | 16.154 | < 0.001 | 10.768 | 1.2234e-10 | COV-D - COV-L; COV-D - COV-M; COV-D - HEA; COV-L - COV-M; COV-L - HEA; Other - COV-M; Other - HEA |
| **L-LDL-TG** | 15.705 | < 0.001 | 10.48 | 2,276E-07 | COV-D - COV-L; COV-D - COV-M; COV-D - HEA; COV-L - COV-M; COV-L - HEA; Other - COV-M; Other - HEA |
| **XS-VLDL-TG** | 15.482 | < 0.001 | 10.337 | 3,044E-07 | COV-D - COV-L; COV-D - COV-M; COV-D - HEA; COV-D - Other; COV-L - COV-M; COV-L - HEA; Other - COV-M; Other - HEA |
| **LDL-TG** | 15.004 | < 0.001 | 10.028 | 5,972E-07 | COV-D - COV-L; COV-D - COV-M; COV-D - HEA; COV-L - COV-M; COV-L - HEA; Other - COV-M; Other - HEA |
| **His** | 14.965 | < 0.001 | 10.003 | 6.1042e-10 | COV-M - COV-D; HEA - COV-D; COV-M - COV-L; HEA - COV-L; COV-M - Other; HEA - Other |
| **M-LDL-TG** | 13.969 | < 0.001 | 9.3522 | 2,636E-06 | COV-D - COV-L; COV-D - COV-M; COV-D - HEA; COV-L - COV-M; COV-L - HEA; Other - COV-M; Other - HEA |
| **S-LDL-TG** | 11.577 | < 0.001 | 7.7558 | 1,006E-04 | COV-D - COV-L; COV-D - COV-M; COV-D - HEA; COV-L - COV-M; COV-L - HEA; Other - COV-M; Other - HEA |
| **XS-VLDL-PL** | 11.112 | < 0.001 | 7.4404 | 2.0125e-07 | COV-D - COV-L; COV-D - COV-M; COV-D - HEA; COV-L - COV-M; COV-L - HEA; Other - COV-M; Other - HEA |
| **Phe** | 10.62 | < 0.001 | 7.1039 | 4.2313e-07 | COV-D - COV-L; COV-D - COV-M; COV-D - HEA; COV-D - Other; COV-L - COV-M; COV-L - HEA |
| **XXL-VLDL-PL** | 10.343 | < 0.001 | 6.9134 | 6.3623e-07 | COV-D - COV-L; COV-D - COV-M; COV-D - HEA; COV-D - Other; COV-L - COV-M; COV-L - HEA; COV-L - Other |
| **Unsaturation** | 9.9304 | < 0.001 | 6.6289 | 1.1735e-06 | COV-L - COV-D; COV-M - COV-D; HEA - COV-D; COV-L - Other; COV-M - Other; HEA - Other |
| **XXL-VLDL-CE** | 9.9203 | < 0.001 | 6.622 | 1.1735e-06 | COV-D - COV-L; COV-D - COV-M; COV-D - HEA; COV-D - Other; COV-L - COV-M; COV-L - HEA |
| **XXL-VLDL-C** | 9.8139 | < 0.001 | 6.5483 | 1.3518e-06 | COV-D - COV-L; COV-D - COV-M; COV-D - HEA; COV-D - Other; COV-L - COV-M; COV-L - HEA |
| **LDL size** | 9.7156 | < 0.001 | 6.4802 | 1.5385e-06 | COV-D - COV-L; COV-D - COV-M; COV-D - HEA; COV-L - COV-M; Other - COV-L; HEA - COV-M; Other - COV-M; Other - HEA |
| **XXL-VLDL-P** | 9.6558 | < 0.001 | 6.4388 | 1,648E-03 | COV-D - COV-L; COV-D - COV-M; COV-D - HEA; COV-D - Other; COV-L - COV-M; COV-L - HEA |
| **XXL-VLDL-FC** | 9.2601 | < 0.001 | 6.1637 | 3.0255e-06 | COV-D - COV-L; COV-D - COV-M; COV-D - HEA; COV-D - Other; COV-L - COV-M; COV-L - HEA |
| **Glucose** | 9.078 | < 0.001 | 6.0367 | 3.9519e-06 | COV-D - COV-M; COV-D - HEA; COV-L - COV-M; COV-L - HEA; Other - COV-M |
| **L-HDL-CE** | 9.019 | < 0.001 | 5.9955 | 4.2387e-06 | COV-L - COV-D; COV-M - COV-D; HEA - COV-D; Other - COV-D; COV-M - COV-L; HEA - COV-L; Other - COV-L |
| **HDL-FC** | 8.9943 | < 0.001 | 5.9782 | 4.3061e-06 | COV-L - COV-D; COV-M - COV-D; HEA - COV-D; Other - COV-D; HEA - COV-L |
| **S-VLDL-TG** | 8.8224 | < 0.001 | 5.858 | 5.5474e-06 | COV-D - COV-M; COV-D - HEA; COV-D - Other; COV-L - COV-M; COV-L - HEA |
| **XL-HDL-TG** | 8.0489 | < 0.001 | 5.3142 | 1,896E-02 | COV-D - COV-L; COV-D - COV-M; COV-D - HEA; COV-L - COV-M; COV-L - HEA; Other - COV-M; Other - HEA |
| **L-HDL-C** | 7.9919 | < 0.001 | 5.274 | 2.0339e-05 | COV-L - COV-D; COV-M - COV-D; HEA - COV-D; Other - COV-D; COV-M - COV-L; HEA - COV-L; Other - COV-L |
| **MUFA** | 7.5825 | < 0.001 | 4.9843 | 3.8386e-05 | COV-D - COV-M; COV-D - HEA; COV-L - COV-M; COV-L - HEA; Other - COV-M |
| **XS-VLDL-L** | 7.5754 | < 0.001 | 4.9793 | 3.8386e-05 | COV-D - COV-L; COV-D - COV-M; COV-D - HEA; COV-L - COV-M; COV-L - HEA; Other - COV-M |
| **Citrate** | 7.5266 | < 0.001 | 4.9447 | 4.0703e-05 | COV-D - COV-L; COV-M - COV-L; HEA - COV-L |
| **S-HDL-TG** | 7.4763 | < 0.001 | 4.909 | 4.3287e-05 | COV-D - COV-L; COV-D - COV-M; COV-D - HEA; COV-D - Other; COV-L - COV-M; COV-L - HEA |
| **XS-VLDL-FC** | 7.2891 | < 0.001 | 4.776 | 5.7616e-05 | COV-D - COV-L; COV-D - COV-M; COV-D - HEA; COV-L - COV-M; COV-L - HEA; Other - COV-M |
| **Tyr** | 7.2308 | < 0.001 | 4.7346 | 6.2141e-05 | COV-D - COV-L; COV-D - COV-M; COV-D - HEA; COV-D - Other |
| **L-HDL-L** | 7.1433 | < 0.001 | 4.6723 | 6.9886e-05 | COV-M - COV-D; HEA - COV-D; Other - COV-D; COV-M - COV-L; HEA - COV-L; Other - COV-L |
| **XXL-VLDL-L** | 7.1356 | < 0.001 | 4.6669 | 6.9886e-05 | COV-D - COV-M; COV-D - HEA; COV-D - Other; COV-L - COV-M; COV-L - HEA; COV-L - Other |
| **XS-VLDL-P** | 7.0512 | < 0.001 | 4.6068 | 7.7517e-05 | COV-D - COV-M; COV-D - HEA; COV-L - COV-M; COV-L - HEA; Other - COV-M |
| **S-HDL-FC** | 7.0498 | < 0.001 | 4.6058 | 7.7517e-05 | COV-L - COV-D; COV-M - COV-D; HEA - COV-D; HEA - COV-L; HEA - COV-M; HEA - Other |
| **L-HDL-P** | 6.8871 | < 0.001 | 4.4898 | 9.8519e-05 | COV-M - COV-D; HEA - COV-D; Other - COV-D; COV-M - COV-L; HEA - COV-L; Other - COV-L |
| **L-HDL-PL** | 6.8819 | < 0.001 | 4.4861 | 9.8519e-05 | COV-M - COV-D; HEA - COV-D; Other - COV-D; COV-M - COV-L; HEA - COV-L; Other - COV-L |
| **ApoB/ApoA1** | 6.8105 | < 0.001 | 4.4352 | 0.00010887 | COV-D - COV-L; COV-D - COV-M; COV-D - HEA; COV-L - COV-M; COV-L - HEA |
| **Pyruvate** | 6.6183 | < 0.001 | 4.298 | 0.0001468 | COV-D - COV-L; COV-D - COV-M; COV-D - HEA; COV-L - HEA; Other - HEA |
| **S-VLDL-P** | 6.2713 | < 0.001 | 4.0498 | 0.00025564 | COV-D - COV-M; COV-D - HEA; COV-L - COV-M; COV-L - HEA |
| **Total-TG** | 6.0066 | < 0.001 | 3.8601 | 0.00038916 | COV-D - COV-M; COV-D - HEA; COV-L - COV-M; COV-L - HEA |
| **Omega-6/Omega-3** | 5.8733 | < 0.001 | 3.7644 | 0.00047721 | COV-D - COV-L; COV-M - COV-L; HEA - COV-L; Other - COV-L |
| **Omega-3** | 5.7704 | < 0.001 | 3.6906 | 0.00055669 | COV-L - COV-M; COV-L - HEA; COV-L - Other |
| **Phosphatidylc** | 5.6938 | < 0.001 | 3.6356 | 0.00062196 | COV-L - COV-D; COV-M - COV-D; HEA - COV-D; HEA - COV-L; HEA - Other |
| **Total-CE** | 5.3898 | < 0.001 | 3.4172 | 0.0010126 | COV-L - COV-D; COV-M - COV-D; HEA - COV-D; HEA - COV-L |
| **S-VLDL-L** | 5.2682 | < 0.001 | 3.3297 | 0.0012197 | COV-D - COV-M; COV-D - HEA; COV-L - COV-M; COV-L - HEA |
| **XL-VLDL-PL** | 5.2239 | < 0.001 | 3.2979 | 0.0012928 | COV-D - COV-M; COV-D - HEA; COV-D - Other; COV-L - COV-M; COV-L - HEA |
| **XXL-VLDL-TG** | 4.9117 | < 0.001 | 3.0734 | 0.0021362 | COV-D - COV-M; COV-D - HEA; COV-D - Other; COV-L - COV-M; COV-L - HEA; COV-L - Other |
| **L-LDL-FC** | 4.8952 | < 0.001 | 3.0615 | 0.0021567 | COV-L - COV-D; COV-M - COV-D; HEA - COV-D; HEA - COV-L |
| **XL-VLDL-P** | 4.8884 | < 0.001 | 3.0566 | 0.0021567 | COV-D - COV-M; COV-D - HEA; COV-D - Other; COV-L - COV-M; COV-L - HEA |
| **XL-VLDL-FC** | 4.8128 | < 0.001 | 3.0023 | 0.0024099 | COV-D - COV-M; COV-D - HEA; COV-D - Other; COV-L - COV-M; COV-L - HEA |
| **L-HDL-FC** | 4.7892 | 0.001 | 2.9853 | 0.002471 | COV-M - COV-D; HEA - COV-D; Other - COV-D; HEA - COV-L; Other - COV-L |
| **XL-HDL-FC** | 4.6062 | 0.0014 | 2.8537 | 0.0032998 | COV-D - COV-M; COV-D - HEA; COV-L - HEA; Other - COV-L; Other - COV-M; Other - HEA |
| **VLDL-P** | 4.5298 | 0.0015 | 2.7989 | 0.0036508 | COV-D - COV-M; COV-D - HEA; COV-L - COV-M; COV-L - HEA |
| **LDL-FC** | 4.5287 | 0.0015 | 2.7981 | 0.0036508 | COV-L - COV-D; COV-M - COV-D; HEA - COV-D; HEA - COV-L |
| **DHA** | 4.499 | 0.0016 | 2.7767 | 0.0037848 | COV-L - COV-D; COV-L - COV-M; COV-L - HEA; COV-L - Other |
| **IDL-CE** | 4.4505 | 0.0018 | 2.7419 | 0.0040474 | COV-L - COV-D; COV-M - COV-D; HEA - COV-D; HEA - COV-L |
| **Cholines** | 4.4189 | 0.0019 | 2.7192 | 0.0042097 | COV-L - COV-D; COV-M - COV-D; HEA - COV-D; HEA - COV-L |
| **L-LDL-C** | 4.3773 | 0.0020 | 2.6893 | 0.0044525 | COV-L - COV-D; COV-M - COV-D; HEA - COV-D |
| **S-VLDL-CE** | 4.3671 | 0.0020 | 2.6819 | 0.0044609 | COV-D - COV-M; COV-D - HEA; COV-L - COV-M |
| **VLDL-PL** | 4.361 | 0.0021 | 2.6776 | 0.0044609 | COV-D - COV-M; COV-D - HEA; COV-L - COV-M |
| **M-LDL-FC** | 4.3061 | 0.0023 | 2.6382 | 0.0048254 | COV-L - COV-D; COV-M - COV-D; HEA - COV-D |
| **L-LDL-CE** | 4.2407 | 0.0025 | 2.5912 | 0.0053116 | COV-L - COV-D; COV-M - COV-D; HEA - COV-D |
| **Acetoacetate** | 4.166 | 0.0028 | 2.5376 | 0.0059379 | COV-L - COV-D; COV-L - COV-M; COV-L - HEA; Other - COV-M; Other - HEA |
| **Total-C** | 4.0398 | 0.0035 | 2.4472 | 0.0072266 | COV-L - COV-D; COV-M - COV-D; HEA - COV-D; HEA - COV-L |
| **LA** | 4.0118 | 0.0037 | 2.4271 | 0.0074805 | COV-M - COV-D; HEA - COV-D; HEA - COV-L |
| **XL-VLDL-L** | 3.9997 | 0.0038 | 2.4185 | 0.007543 | COV-D - COV-M; COV-D - HEA; COV-D - Other; COV-L - COV-M; COV-L - HEA |
| **Lactate** | 3.9484 | 0.0041 | 2.3818 | 0.008115 | COV-D - COV-L; COV-M - COV-L; Other - COV-L |
| **SFA** | 3.7687 | 0.0055 | 2.2532 | 0.010788 | COV-D - COV-M; COV-L - COV-M; Other - COV-M |
| **Ile** | 3.7553 | 0.0057 | 2.2436 | 0.010906 | COV-L - COV-D; COV-L - COV-M; COV-L - Other |
| **L-VLDL-PL** | 3.7468 | 0.0057 | 2.2376 | 0.010937 | COV-D - COV-M; COV-D - HEA; COV-L - COV-M |
| **VLDL-TG** | 3.7348 | 0.0059 | 2.229 | 0.011035 | COV-D - COV-M; COV-D - HEA; COV-L - COV-M; COV-L - HEA |
| **Phosphoglyc** | 3.6581 | 0.0066 | 2.1743 | 0.012381 | COV-L - COV-D; HEA - COV-D; HEA - COV-L |
| **HDL-TG** | 3.5888 | 0.0075 | 2.1248 | 0.013677 | COV-D - COV-L; COV-D - COV-M; COV-D - HEA; COV-D - Other |
| **LDL-C** | 3.5845 | 0.0075 | 2.1218 | 0.013677 | COV-L - COV-D; HEA - COV-D |
| **XL-VLDL-C** | 3.5726 | 0.0077 | 2.1133 | 0.013803 | COV-D - COV-M; COV-D - HEA; COV-L - COV-M |
| **XL-VLDL-TG** | 3.5198 | 0.0084 | 2.0757 | 0.014896 | COV-D - COV-M; COV-L - COV-M; COV-L - HEA; COV-L - Other |
| **L-VLDL-FC** | 3.5002 | 0.0086 | 2.0618 | 0.015224 | COV-D - COV-M; COV-D - HEA; COV-L - COV-M |
| **VLDL-L** | 3.4728 | 0.0090 | 2.0423 | 0.015763 | COV-D - COV-M; COV-D - HEA; COV-L - COV-M |
| **Omega-6** | 3.364 | 0.0108 | 1.9649 | 0.018646 | COV-L - COV-D; COV-M - COV-D; HEA - COV-D; HEA - COV-L |
| **IDL-C** | 3.3131 | 0.0117 | 1.9288 | 0.020062 | COV-L - COV-D; COV-M - COV-D; HEA - COV-D; HEA - COV-L |
| **LDL-CE** | 3.2815 | 0.0124 | 1.9064 | 0.020764 | COV-L - COV-D; HEA - COV-D |
| **Total-PL** | 3.28 | 0.0124 | 1.9054 | 0.020764 | COV-L - COV-D; HEA - COV-D; HEA - COV-L |
| **S-VLDL-C** | 3.2112 | 0.0139 | 1.8566 | 0.023007 | COV-D - COV-M; COV-L - COV-M |
| **S-VLDL-PL** | 3.1736 | 0.0147 | 1.8301 | 0.024225 | COV-D - COV-M; COV-L - COV-M |
| **L-VLDL-P** | 3.1495 | 0.0153 | 1.813 | 0.024766 | COV-D - COV-M; COV-L - COV-M |
| **S-LDL-FC** | 3.1478 | 0.0154 | 1.8118 | 0.024766 | COV-L - COV-D; HEA - COV-D |
| **Ala** | 3.1428 | 0.0155 | 1.8083 | 0.024766 | COV-D - Other; COV-L - Other; COV-M - Other; HEA - Other |
| **VLDL-FC** | 3.0483 | 0.0181 | 1.7416 | 0.028607 | COV-D - COV-M; COV-L - COV-M |
| **XL-HDL-PL** | 3.0339 | 0.0185 | 1.7315 | 0.029019 | Other - COV-D; Other - COV-L; Other - COV-M; Other - HEA |
| **L-VLDL-C** | 3.0224 | 0.0189 | 1.7234 | 0.029296 | COV-D - COV-M; COV-L - COV-M |
| **XL-HDL-L** | 2.9505 | 0.0212 | 1.6728 | 0.032625 | Other - COV-D; Other - COV-L; Other - COV-M; Other - HEA |
| **Clinical LDL-C** | 2.884 | 0.0236 | 1.6261 | 0.036004 | COV-L - COV-D; HEA - COV-D |
| **XL-HDL-CE** | 2.8002 | 0.0270 | 1.5674 | 0.040648 | Other - COV-D; Other - COV-L; Other - COV-M; Other - HEA |
| **XL-HDL-C** | 2.7979 | 0.0271 | 1.5658 | 0.040648 | Other - COV-D; Other - COV-L; Other - COV-M; Other - HEA |
| **Total-FA** | 2.7389 | 0.0298 | 1.5246 | 0.044226 | COV-D - COV-M; COV-L - COV-M |
| **M-LDL-C** | 2.7347 | 0.0300 | 1.5217 | 0.044226 | COV-L - COV-D; HEA - COV-D |
| **XL-VLDL-CE** | 2.7097 | 0.0313 | 1.5042 | 0.045647 | COV-D - COV-M; COV-L - COV-M |

Table S3: Area under the curve, p value, and log2 fold change of forty-nine metabolites related to the hospital risk stratification of COVID-19 patients.

| **Metabolites** | **AUC** | **Pval** | **FC** |
| --- | --- | --- | --- |
| Albumin | 0.97 | < 0.001 | -0.5756 |
| GlycA | 0.91 | < 0.001 | 0.4186 |
| L-LDL-TG | 0.88 | < 0.001 | 0.7248 |
| LDL-TG | 0.88 | < 0.001 | 0.7290 |
| His | 0.88 | < 0.001 | -0.6185 |
| M-LDL-TG | 0.88 | < 0.001 | 0.7739 |
| IDL-TG | 0.86 | < 0.001 | 0.6604 |
| XS-VLDL-TG | 0.85 | < 0.001 | 0.7008 |
| Acetoacetate | 0.84 | 0.009 | 2.84 |
| Glucose | 0.84 | < 0.001 | 0.6499 |
| S-LDL-TG | 0.83 | < 0.001 | 4.5861 |
| PUFA/MUFA | 0.79 | < 0.001 | -0.3074 |
| XS-VLDL-PL | 0.78 | < 0.001 | 0.5142 |
| MUFA | 0.78 | < 0.001 | 0.4389 |
| S-VLDL-TG | 0.77 | < 0.001 | 0.6489 |
| TG/PG | 0.76 | < 0.001 | 0.4545 |
| Omega-6/Omega-3 | 0.76 | < 0.001 | -0.5658 |
| M-HDL-CE | 0.76 | < 0.001 | -0.3835 |
| 3HB | 0.76 | 0.0292 | 2.0148 |
| S-HDL-CE | 0.74 | < 0.001 | -0.2413 |
| M-HDL-C | 0.74 | < 0.001 | -0.3711 |
| Omega-3 | 0.74 | < 0.001 | 0.8271 |
| S-VLDL-P | 0.74 | < 0.001 | 4.1361 |
| XXL-VLDL-CE | 0.73 | < 0.001 | 0.9682 |
| XS-VLDL-L | 0.73 | < 0.001 | 0.4371 |
| M-HDL-P | 0.73 | < 0.001 | -0.3556 |
| HDL-CE | 0.73 | < 0.001 | -0.3085 |
| XS-VLDL-P | 0.72 | < 0.001 | 0.4395 |
| S-VLDL-L | 0.72 | < 0.001 | 0.5516 |
| XS-VLDL-FC | 0.72 | < 0.001 | 0.4376 |
| HDL-P | 0.72 | < 0.001 | -0.2305 |
| SFA | 0.72 | < 0.001 | 0.3205 |
| ApoA1 | 0.72 | < 0.001 | -0.2053 |
| ApoB/ApoA1 | 0.72 | < 0.001 | 0.4775 |
| XL-HDL-TG | 0.72 | 0.0012 | 0.4872 |
| Total-TG | 0.72 | < 0.001 | 0.5276 |
| Citrate | 0.71 | 0.0012 | -0.2374 |
| XXL-VLDL-C | 0.71 | < 0.001 | 0.8155 |
| M-HDL-L | 0.71 | < 0.001 | -0.2800 |
| XXL-VLDL-P | 0.71 | < 0.001 | 0.8397 |
| S-VLDL-CE | 0.71 | < 0.001 | 0.5293 |
| S-HDL-TG | 0.71 | < 0.001 | 0.3863 |
| DHA | 0.71 | < 0.001 | 0.6649 |
| XXL-VLDL-PL | 0.71 | < 0.001 | 0.5444 |
| Lactate | 0.71 | 0.0077 | -0.4003 |
| S-HDL-C | 0.70 | 0.0013 | -0.1803 |
| HDL-C | 0.70 | < 0.001 | -0.2662 |
| M-HDL-PL | 0.70 | < 0.001 | -0.2387 |
| S-HDL-P | 0.70 | 0.0014 | -0.1766 |
